# A Morning-Specific Phytohormone Gene Expression Program underlying Rhythmic Plant Growth

**DOI:** 10.1371/journal.pbio.0060225

**Published:** 2008-09-16

**Authors:** Todd P Michael, Ghislain Breton, Samuel P Hazen, Henry Priest, Todd C Mockler, Steve A Kay, Joanne Chory

**Affiliations:** 1 Plant Biology Laboratory, The Salk Institute for Biological Studies, La Jolla, California, United States of America; 2 Section of Cell and Developmental Biology, University of California San Diego, La Jolla, California, United States of America; 3 Department of Botany and Plant Pathology and Center for Genome Research and Biocomputing, Oregon State University, Corvallis, Oregon, United States of America; 4 Howard Hughes Medical Institute, The Salk Institute for Biological Studies, La Jolla, California, United States of America; Max Planck Institute for Developmental Biology, Germany

## Abstract

Most organisms use daily light/dark cycles as timing cues to control many essential physiological processes. In plants, growth rates of the embryonic stem (hypocotyl) are maximal at different times of day, depending on external photoperiod and the internal circadian clock. However, the interactions between light signaling, the circadian clock, and growth-promoting hormone pathways in growth control remain poorly understood. At the molecular level, such growth rhythms could be attributed to several different layers of time-specific control such as phasing of transcription, signaling, or protein abundance. To determine the transcriptional component associated with the rhythmic control of growth, we applied temporal analysis of the Arabidopsis thaliana seedling transcriptome under multiple growth conditions and mutant backgrounds using DNA microarrays. We show that a group of plant hormone-associated genes are coexpressed at the time of day when hypocotyl growth rate is maximal. This expression correlates with overrepresentation of a *cis*-acting element (CACATG) in phytohormone gene promoters, which is sufficient to confer the predicted diurnal and circadian expression patterns in vivo. Using circadian clock and light signaling mutants, we show that both internal coincidence of phytohormone signaling capacity and external coincidence with darkness are required to coordinate wild-type growth. From these data, we argue that the circadian clock indirectly controls growth by permissive gating of light-mediated phytohormone transcript levels to the proper time of day. This temporal integration of hormone pathways allows plants to fine tune phytohormone responses for seasonal and shade-appropriate growth regulation.

## Author Summary

In plants, stems elongate faster at dawn. This time-of-day–specific growth is controlled by integration of environmental cues and the circadian clock. The specific effectors of growth in plants are the phytohormones: auxin, ethylene, gibberellins, abscisic acid, brassinosteroids, and cytokinins. Each phytohormone plays an independent as well as an overlapping role in growth, and understanding the interactions of the phytohormones has dominated plant research over the past century. The authors present a model in which the circadian clock coordinates growth by synchronizing phytohormone gene expression at dawn, allowing a plant to control growth in a condition-specific manner. Furthermore, the results presented provide a new framework for future experiments aimed at understanding the integration and crosstalk of the phytohormones.

## Abstract 3

Why do plants grow faster at dawn? New results suggest that light and the circadian clock coordinate growth by synchronizing the expression of plant hormone genes at dawn.

## Introduction

Plant growth involves the coordination of cell division and expansion, which is the result of developmental programs initiated by both intrinsic and extrinsic factors. Of the various environmental parameters that regulate plant development, light quality, quantity, and duration play important roles. In dark-grown dicotyledonous seedlings such as Arabidopsis thaliana, the embryonic stem or hypocotyl grows rapidly by longitudinal cell expansion in a process referred to as hypocotyl elongation. At the same time, the embryonic leaves, or cotyledons, remain small and unexpanded. In contrast, the rate of hypocotyl elongation is inhibited and cotyledon expansion is promoted by light [1]. In mature plants, a similar differential growth process occurs when light is limiting or in response to shade, whereby stems and petioles elongate at the expense of leaf expansion [2]. However, it remains unclear how the plant regulates these distinct growth states.

Differential growth responses are executed by small-molecule hormones, also called phytohormones, that are synthesized and transported throughout the plant [3]. At least six classes of phytohormones nonredundantly control growth [1]. Gibberellins (GA), auxin (IAA), and brassinosteroids (BR) promote cell expansion along longitudinal axes, and abscisic acid (ABA) antagonizes both GA- and BR-regulated growth; whereas cytokinin (CK) and ethylene (referred to here as ACC) promote cell expansion along transverse axes [4]. One apparent paradox regarding how phytohormones orchestrate growth programs revolves around their simultaneous redundancy and specificity. As an example, mutations in biosynthetic enzymes of either GA or BR result in severe dwarf phenotypes, consistent with these pathways acting nonredundantly [3]. This is also consistent with recent analysis of global gene expression profiles from seedlings treated with specific phytohormones, which suggests that hormonal pathways do not converge on a core early transcriptional growth-regulatory module, but instead regulate distinct target genes [4]. In contrast, BR and IAA treatments result in the up-regulation of many common target genes, suggesting that there can be significant integration and crosstalk between these hormone pathways [5]. Finally, one model suggests that a family of nuclear-localized proteins, identified from GA response pathway mutations, acts as a central transcriptional integrator of growth controlling pathways [6]. How these pathways are coordinated to achieve an optimal body plan for a particular environment is currently a key question in plant development.

It is known that phytohormone levels vary over the course of the day. Bioactive phytohormone levels of BR, IAA, ACC, GA, and ABA accumulate from dawn to midday under light/dark cycles [7–12]. In addition, either light/dark cycles or the circadian clock have been shown to regulate certain phytohormone-associated transcripts [7,9,13,14], suggesting that there is an intimate connection between phytohormone activity and time-of-day specificity. The circadian clock and light/dark cycles interact to control daily hypocotyl growth through the activity of the transcription factors phytochrome interacting factor 4 (PIF4) and PIF5 [15–18]. In addition, the circadian clock gates both IAA pathways and IAA-mediated growth [14]. However, the mechanisms defining the relationship between the circadian clock, light signaling, and phytohormone-controlled growth remain unclear.

Organisms have evolved circadian clocks with internal periods of about 24 h/ that allow synchrony with their external environment [19,20]. Plants with circadian period lengths that match that of their environment display enhanced fitness because they are able to correctly phase key metabolic and physiological events relative to the daily changes in the environment, such as the light/dark cycle [21]. Underlying the ability to properly anticipate and respond to daily changes in the environment is an extensive transcriptional network governed by the circadian clock, light, and temperature cycles, which ensures that almost 90% of transcripts in *Arabidopsis* accumulate at specific times over the day [19,22]. Since growth is time-of-day specific, we reasoned that a temporal integration of a transcriptional component of the phytohormone pathways could be part of the specificity and redundancy.

Here, we find that the circadian clock and light signaling pathways interact to coordinate the expression of biosynthetic, catabolic, receptor, and signaling genes from multiple phytohormone pathways. The coordination of phytohormone transcript abundance correlated well with the time of maximum growth, consistent with phytohormone pathways directly controlling growth. We identified and characterized a *cis*-regulatory element that is overrepresented in phytohormone gene promoters and showed that it confers both diurnal and circadian cycling to the luciferase (LUC) reporter in vivo. On the basis of our observations, we propose a model in which the circadian clock indirectly controls growth by maintaining light signaling during the early part of the evening, which ensures that peak phytohormone transcript abundance coincides with the end of the dark period. Our findings provide a framework for understanding how seedlings transition from dark-dependent to light-dependent growth after germination and respond appropriately to both acute and long-term changes in the light environment.

## Results

### Phytohormone Gene Expression Correlates with Growth

Based on reports that phytohormone abundance changes over the day and the observation that there is time-of-day–specific hypocotyl growth, we hypothesized that genes involved in the generation and action of phytohormones might also be regulated in a time-of-day fashion. To test this hypothesis, we first assembled a list of 182 “phytohormone genes” from the literature, that represent six phytohormone pathways: ABA, ACC, BR, CK, GA, and IAA (Table S1). Since our goal was to focus on the generation and action of phytohormones, we chose the phytohormone genes based on genetic and expression data implicating them in biosynthesis, catabolism, receptor, and signaling processes. The phytohormone gene list should be considered a tool rather than representing an exhaustive inventory of all known phytohormone genes.

**Figure 1:**
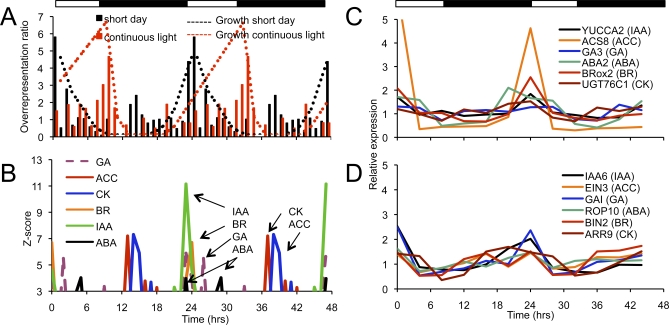
Phytohormone Transcript Abundance Correlates with Time of Hypocotyl Growth under Short-Day Photocycles and Continuous Light (A) Phytohormone genes display peak transcript overrepresentation during the phase of peak growth. Peak growth rate under short-day photocycles (black dashed lines) and continuous light (red dashed lines) reproduced from [15–17] correlates with peak transcript overrepresentation under short-day photocycles (black bars) and continuous light (red bars). (B) *Z*-score significance score for individual groups of phytohormone genes under short day. Data generated using PHASER. *Z*-score = 3, approximately *p* = 0.01. GA (dashed brown), ACC (red), CK (blue), BR (orange), IAA (green), and ABA (black). (C) Phytohormone biosynthesis transcripts show peak abundance during the growth phase under short-day photocycles. (D) Phytohormone signaling transcripts show peak abundance during the growth phase under short-day photocycles. Data in (A) and (B) are double plotted for visualization purposes; 1 d (24 h) of data copied and graphed a second day to allow visual continuity of the time-of-day data. Gene names in (C) and (D) are indicated, and the phytohormone pathway is in parentheses.

Since it has been shown that growth is maximal at the dark-to-light transition (dawn) under short-day photocycles (8-h light/16-h dark) and light-to-dark transition (dusk) under circadian conditions of continuous light and temperature [15–18], we asked whether the phytohormone genes are overrepresented during these times of day. We developed a phase overepresentation graphing tool to help us determine the statistical significance of any observed enrichment at a specific time of day. This tool works by calculating the number of genes exhibiting peak expression at a particular time of day versus the number expected, and statistics are derived by permutation. We developed a Web interface called PHASER that enables any gene set to be searched for a time-of-day coexpression signature (http://phaser.cgrb.oregonstate.edu). Using our phytohormone gene list, we identified a highly significant overrepresentation (*p* < 0.00001) of phytohormone genes 1 h before (zeitgeber time 23; ZT23) and 1 h after dawn (ZT0) under short-day photocycles. Likewise, we found a similar overrepresentation (*p* < 0.00001) at subjective dusk under circadian conditions (circadian time, CT8 and 9; Figure 1A). The correlation between the time of maximum growth and overrepresentation of peak phytohormone transcript abundance suggested to us that there may be a connection between these two activities (Figure 1A, dotted lines) [15–18].

The shift from dawn to subjective dusk in maximum growth rate between short-day and circadian conditions [15] most likely reflects a resetting of the circadian clock associated with the release into continuous light after growth under light/dark cycles. When the plant experiences the first day in continuous light, the extended light period may be interpreted as a long day, and the phase of the circadian clock is reset, resulting in a “subjective” phase delay in growth rate and the observed phytohormone transcript abundance [19,22].

Under short-day photocycles, we noted a small group of genes overrepresented after dusk at ZT13, 14 (*p* < 0.01; Figure 1A and 1B). To find out whether the different clusters of phytohormone genes were due to a specific phytohormone group, we constructed phase overrepresentation plots for each individual class of phytohormone genes, and plotted their corresponding *Z*-scores (Figure 1B). When each phytohormone class was evaluated separately under short-day photocycles, CK and ACC genes were overrepresented during the dark period, whereas BR, IAA, GA, and ABA genes were overrepresented at or around dawn (Figure 1B). These results most likely reflect the distinct roles of these classes of phytohormone genes in transverse and longitudinal cell growth, respectively. We have focused on the phytohormone genes that are correlated with maximal elongation rate, which are the genes that display peak abundance at or around dawn.

**Figure 2:**
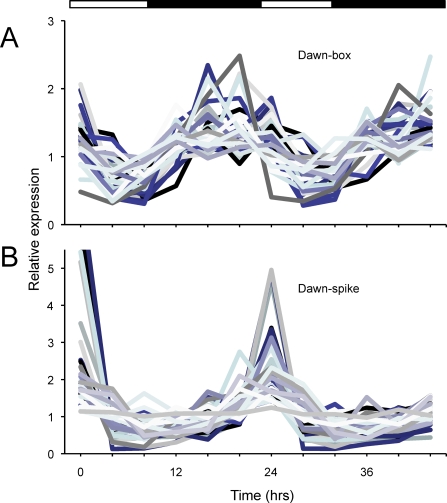
Phytohormone Genes Associated with Growth Phase Of the 182 phytohormone genes, 71 were identified as growth associated (*p* < 0.01), using either dawn-box (A) or dawn-spike (B) models under short-day photocycles. (A) Thirty-one dawn-box growth-associated phytohormone genes. (B) Forty dawn-spike growth-associated phytohormone genes.

While visually inspecting the expression patterns of the phytohormone genes, we identified two patterns of expression that correlated well with maximum growth over the day: genes that peaked directly at dawn, such as the biosynthesis (Figure 1C) and catabolism genes, and genes that increased during the dark period, such as the signaling (Figure 1D) and receptor genes. We summarized these two growth-associated patterns as “dawn-spike” and “dawn-box,” respectively, and chose two genes from each group as “models” to interrogate the 182 phytohormone genes for similar patterns of expression. Seventy-one genes had significant correlations with the growth-associated patterns (*p* < 0.01; 31 dawn-box and 40 dawn-spike), and we focused on these genes in all subsequent analyses (Figure 2 and Table S2).

### Both the Downstream Targets of Phytohormone Pathways and Phytohormone Genes Are Coexpressed

**Figure 3:**
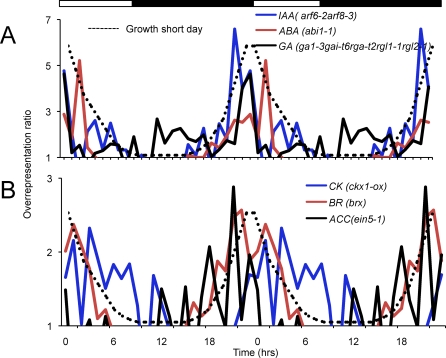
Misregulated Genes in Selected Phytohormone Mutants Are Expressed at Dawn under Short-Day Photocycles (A) The genes that are differentially expressed in the *arf6-2arf8-3*, *abi1-1*, and *DELLApenta* (*ga1-3 gai-t6 rga-t2 rgl1-1 rgl2-1*) mutants are overrepresented around dawn under short-day photocycles. (B) The genes that are differentially expressed in the *ckx1-ox*, *brx*, and *ein5-1* mutants are overrepresented around dawn under short-day photocycles. Mutant microarray data from published sources and differentially expressed genes (*p* < 0.01) were identified by comparing mutant expression to the wild-type expression (Materials and Methods). *Z*-score profile is double plotted for visualization purposes, and the dotted line is the growth rate under short-day photocycles. Hypocotyl growth rate under short-day photocycles (black dotted line) is reproduced to provide a frame of reference for time of day of maximal hypocotyl growth.

It has been shown that some hormone-responsive genes also cycle over the day [23]. We reasoned that if phytohormone pathways are active during the growth phase of the day, then phytohormone mutants should preferentially affect genes that are normally expressed during the growth phase. To test this hypothesis, we analyzed the genes affected in selected phytohormone mutants by using publicly available Affymetrix microarray datasets in either the ArrayExpress or the Gene Expression Omnibus (GEO) databases (Materials and Methods). To identify the genes associated with the GA pathway, we looked at the *DELLApenta* (*ga1-3 gai-t6 rga-t2 rgl1-1 rgl2-1*) mutant [24]; for the IAA pathway, the *auxin response factor* (*arf*) *arf6-2 arf8-3* mutant [25]; for the ACC pathway, the *ein5-1* mutant [26]; for the BR pathway, the *brx* mutant [27], for the ABA pathway, the *abscisic acid insensitive 1* (*abi1-1*) mutant; and for the CK pathway, the *ckx1-ox* mutant. We identified the genes that were differentially regulated for each mutant versus wild type (*p* < 0.01, Materials and Methods) and used this gene list to identify a time-of-day signature under short-day photocycles using PHASER (Figure 3). We found that the genes that were differentially regulated in these mutants were expressed at specific times of day under short-day photocycles. The phase of expression of the genes in the ABA, IAA, GA, and BR pathways are closely associated with the time of active growth under short day (Figure 3). These results are consistent with the ABA, IAA, GA, and BR pathways acting through genes that are expressed in the late night or early morning.

### The HUD (CACATG) Element Is Enriched in Phytohormone Gene Promoters

**Figure 4:**
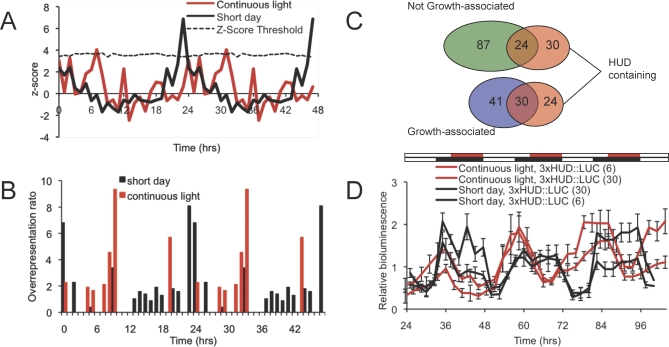
The HUD Motif (CACATG) Confers Diurnal and Circadian Regulation (A) The HUD motif (CACATG) is overrepresented in promoter of cycling genes expressed (*Z*-score, significance score) at midday under continuous light (red) and dawn under short-day photocycles (black) [19]. The *Z*-score profile is double plotted for visualization purposes. Black dotted line is the *Z*-score significance threshold (*p* < 0.05). (B) Phase overrepresentation of the 54 phytohormone genes with one or more HUD in their promoter (500 bp) under short-day photocycles (black) or continuous light (red). (C) Venn diagram of the 54 phytohormone genes with at least one HUD in their promoter (500 bp) and either the genes that are not growth associated (111 genes, green) or are growth associated (71 genes, blue). (D) Two independent T3 lines (6 and 30) carrying three tandem HUD repeats fused to LUC and a minimal promoter confer night-specific activity under short-day photocycles (black lines) and evening-specific activity under continuous conditions (red lines). Data represent the average of four to six seedlings from two independent experiments. Error bars represent standard error of the mean.

Our observations suggest that time-of-day–specific growth rate changes may be controlled in part through a coordination of phytohormone transcript abundance. This observation provides a testable hypothesis as to how plants organize their growth programs so that maximal growth rate is restricted to the correct time of day. In a previous study, we identified several *cis*-acting elements with a specific pattern of overrepresentation at different phases of the day. One of these (CACATG) was overrepresented in the promoters of cycling genes whose phase of expression was around dawn under short-day photocycles and subjective dusk under continuous light (Figure 4A) [19]. The CACATG consensus element was shown previously to be overrepresented in genes that respond to both BR and IAA treatment [28], and is related to the Ebox (CANNTG), which is the target of the BR transcription factors BES1 and BIM1 [29]. We named this element Hormone Up at Dawn (HUD).

We searched the list of 71 phytohormone gene promoters (500 bp) for overrepresented words, and found that the HUD was significantly enriched (*Z*-score = 5.2, corrected *p* = 9.8 × 10^−08^; Table S3). Furthermore, the 54 phytohormone genes with the HUD in their promoter were overrepresented at dawn under short-day photocycles and dusk under continuous light (Figure 4B). To display the level of association between the phytohormone gene list and the presence of HUD element, we created a Venn diagram comparing the presence of the HUD in the 71 phytohormone genes associated with growth and the 111 phytohormone genes not associated with growth (Figure 4C).

To test the activity of the HUD element, we analyzed T3 transgenic plants carrying a promoter::luciferase fusion (*3xHUD::LUC*). A multimerized version of the HUD motif was sufficient to confer time-of-day activity to LUC in vivo under both short-day photocycles, as well as in continuous light (Figure 4D). The *3xHUD::LUC* activity fit our prediction well in continuous light with peak LUC activity at dusk. However, the LUC activity deviated from our prediction under short-day photocycles, showing increased activity immediately after the plants experienced darkness. This pattern of expression is reminiscent of previous reports showing that circadian clock mutants grow immediately when moved into the dark, which was attributed to the circadian clock controlling or gating the rate at which light signaling is attenuated during the early evening [15]. This expression pattern in short days suggested to us that although the HUD is sufficient to confer circadian regulation, additional element(s) are required in the HUD-containing promoter context for light-regulated transcription in the early evening.

### The Circadian Clock Modulates the Direct Effects of Light on Growth

To directly test the hypothesis that transcriptional coordination of phytohormone transcript abundance affects hypocotyl growth, we conducted microarray time courses in mutants that have aberrant hypocotyl growth under light/dark cycles: two arrhythmic circadian mutants *lux arrhythmo* (*lux-2*) [30] and *late elongated hypocotyl* (*lhy*) [31], and one red-light photoreceptor mutant, *phytochrome B* (*phyB-9*) [32]. Table S1 provides information about each phytohormone gene examined, including the time of maximum transcript abundance for the cycling gene. In addition, the wild-type time courses confirmed growth-specific expression of the phytohormone genes.

**Figure 5:**
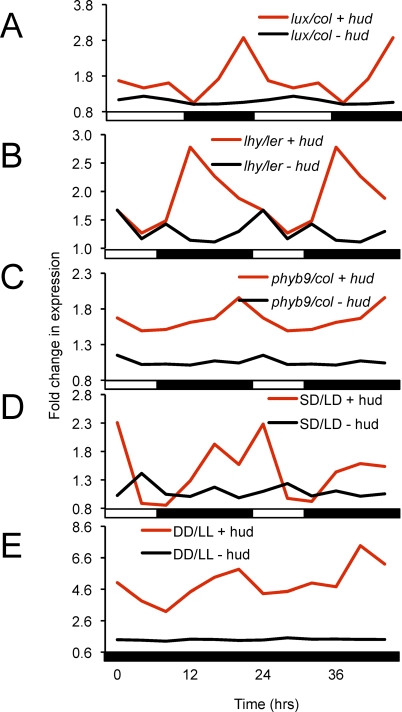
The Growth-Associated Phytohormone Genes Are Linked to Dark-Induced Growth Regulation To determine the time-dependent behavior of the growth-associated phytohormone genes controlled by the HUD element, the average fold increase between mutant and wild type for each time point is presented (red lines). As a control, the average fold changes for the phytohormone genes not associated with growth that do not contain an HUD element are also presented (black lines). In circadian mutants, phytohormone transcript abundance increases during the dark period compared to wild type, whereas it is constantly higher at any time of the day in *phyB* mutant. (A) *lux-2* versus L*er* under intermediate-day photoperiods (12-h light/12-h dark). (B) *lhy* versus Col under short-day photoperiods (8-h light/16-h dark). (C) *phyB-9* versus Col under short-day photoperiods. (D) Short-day versus long-day photocycles. (E) Continuous dark (DD) versus continuous light (LL) [19]. One-day (six time point) time courses are double plotted for visualization purposes. Black bars represent the dark period. Bars at the bottom of each graph represent light/dark cycle.

To examine the behavior of the phytohormone genes in circadian and light signaling mutants with aberrant hypocotyl growth, we assembled two groups of genes, HUD-containing phytohormone genes associated with growth, and genes lacking the HUD that are not associated with growth (30 and 87 genes, respectively, Figure 4C). Because we found that the HUD element was sufficient to confer diurnal and circadian regulation, we reasoned that genes with the HUD element would be preferentially disrupted in the mutants. We compared the average fold change at each time point, between the mutant and its respective wild type, of the HUD-containing genes, with the average fold change between the mutant and its respective wild type in the genes lacking the HUD. As shown in Figure 5A and 5B, there is an increase in expression of the HUD-containing phytohormone genes during the dark phase in the two clock mutants, which is not found in genes lacking the HUD. It appears that phytohormone gene transcript levels increase during the dark period, similar to the pattern of growth in circadian clock mutants [15] and the *3xHUD::LUC* reporter under short-day photocycles. This pattern of expression suggests that the circadian clock acts to control or gate the response of the HUD-containing phytohormone genes so that they are not expressed during the dark phase of the cycle.

In the *phyB* mutant, the HUD-containing phytohormone genes are not directly induced by darkness, but are constantly elevated as compared with the phytohormone genes without the HUD. This suggests that the response to the onset of darkness may be mediated via PHYB signaling pathways. To further test the association between darkness, growth, and the HUD-containing phytohormone genes, we analyzed their expression level in two other sets of time courses. In the first set, short day versus long day, the length of the dark period is twice that of the light period following a photoperiod difference. In the second set, seedlings entrained in the dark or light by temperature cycles were respectively released under continuous dark (DD) or continuous light (LL) conditions to monitor expression. For each of these comparable sets, the HUD-containing phytohormone gene expression is increased as compared to the genes lacking the HUD (Figure 5D and 5E). These results suggest that the expression of the HUD-containing phytohormone genes increases in the absence of light signaling (darkness or the light signaling mutant, *phyB*) and that, in part, the circadian clock specifically acts to abrogate this expression during the early part of the dark period. One explanation is that the circadian clock is required to maintain flux through downstream light signaling pathways in the early part of the night, so despite the plant experiencing darkness, HUD-containing phytohormone gene expression is moderate under long nights.

These results also suggest that the circadian clock acts upstream of light signaling in controlling expression during the dark period of the diurnal cycle. To resolve this, we looked more closely at expression in the *lhy* mutant. LHY is a single MYB transcription factor that acts in the core feedback loop of the circadian clock. It was previously shown in the *lhy* mutant that the peak expression of genes directly controlled by the circadian clock are shifted by 12 h (antiphase) compared to wild type (Figure S2) [30,33]. To quantify the timing change of phytohormone genes, we determined the phase of expression by fitting the dawn models to the *lhy* and parental (Landsberg *erecta*; L*er*) time course. Of the 95 phytohormone genes that significantly matched the models in L*er* (*p* < 0.01), 73 had the same phase in *lhy* (*p* < 0.01), nine were antiphasic to their L*er* counterpart (negative correlation), and 13 were not significantly expressed. In the case in which the circadian clock would have directly controlled phytohormone expression, we would have expected a much higher proportion of genes with antiphasic expression like the core circadian clock genes. Instead, we found that most (77%) of the genes maintained phase with increased expression during the dark phase of the day, consistent with the circadian clock gating expression, and not directly controlling it. We noted that consistent with this interpretation, the expression of circadian clock genes was not affected in a *phyB* mutant, suggesting that PHYB (or light signaling) is downstream of the circadian clock (Figure S1). Taken together, these results support the notion that the circadian clock gates light signaling, which directly controls the phasing of HUD-containing phytohormone genes.

### Light Signaling Directly Controls Growth Phytohormone Gene Expression and Hypocotyl Elongation

**Figure 6:**
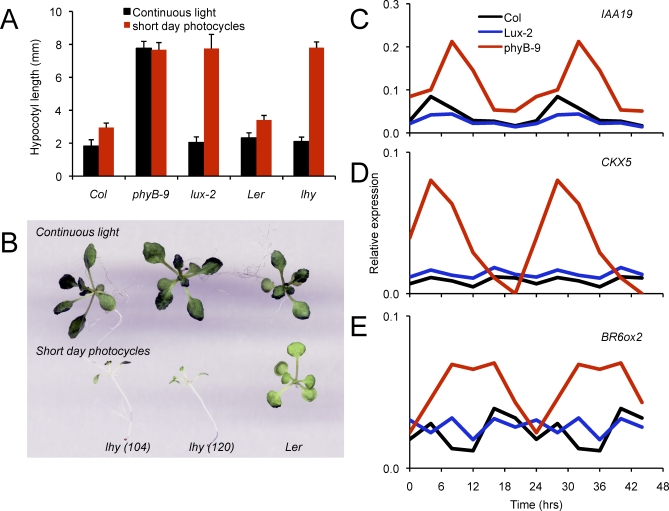
Light Signaling Directly Controls Phytohormone Gene Expression and Growth (A) Continuous light (black bars) rescues the long hypocotyl defect caused under short-day photocycles (red bars) in *lux-2* and *lhy* mutants, but not in the *phyB-9*. Plants were grown under continuous light and thermocycles (12-h 22 °C/12-h 12 °C) to ensure synchronization of the circadian clock for expression studies; results without thermocycles (constant 22 °C) were the same as the results presented here and as those described previously [30,34]. Measurements are an average of at least ten 7-d-old seedlings, and error bars are ± standard deviation. Results represent three independent biological experiments. (B) Continuous light rescues the severe developmental defects of the *lhy* mutants under short-day photocycles. Two alleles of *lhy*, *lhy (104)* and *lhy (120)*, and the parental *L*er were grown as in (A) for 2 wk. The long hypocotyl phenotype of *lhy* under short-day photocycles is completely rescued under continuous light. Results represent three independent biological experiments. (C–E) Continuous light (and thermocycles) as in (A) restores the expression of (C) *IAA19*, (D) *CKX5*, and (E) *BR6ox2* to wild type in *lux-2*, but not in *phyB-9*. The same pattern of expression as in *lux-2* was observed for *lhy* (unpublished data). Expression was measured by qRT-PCR in two independent biological replicates.

Our hypothesis that the light signaling pathway in the dark directly controls growth is further supported by the observation that the long hypocotyl phenotype seen under light/dark cycles in the circadian clock mutants *early flowering 3* (*elf3*), *elf4*, and *lux* can to be rescued if plants are grown under continuous white light [30,34]. To confirm this result with other clock mutants, we showed that under continuous light, both *lhy* and *lux-2* can also be rescued for hypocotyl growth (Figure 6A). Strikingly, we noticed that under short-day photoperiods, *lhy* mutants exhibit an elongated hypocotyl phenotype during its entire life cycle, but in continuous light, it grew essentially as wild type (Figures 6B and S3). In contrast, we could not rescue the long hypocotyl phenotype of *phyB-9* regardless of the light condition (Figure 6A), suggesting that hypocotyl defects can be uncoupled from circadian dysfunction, but not from the loss of PHYB activity or a period of darkness such as in short-day photoperiod.

To test whether the rescue of hypocotyl growth in clock mutant and the long hypocotyl of *phyB-9* could be associated with phytohormone transcript abundance, we measured the level of phytohormone gene expression in *lux-2*, *lhy*, and *phyB-9* under continuous light using quantitative real-time PCR (qRT-PCR). Expression of *IAA19*, *CKX5*, and *BR6ox2* were restored to wild-type levels in *lux-2* and *lhy*, whereas they remained higher in *phyB-9*, which in all cases is in accordance with the observed hypocotyl growth phenotype (Figure 6C–6E). These results provide further evidence for a functional association between the transcription module that we identified and the control of hypocotyl growth rate. They also demonstrate that darkness, or the loss of light signaling (such as in *phyB-9*), is important in the control of phytohormone transcript abundance, whereas the circadian clock must play an indirect role, perhaps through modulation of light signaling. Finally, these results may explain why arrhythmic circadian clock mutants do not have hypocotyl defects under continuous white light, yet have very long hypocotyls under light/dark cycles [30,34].

### Shade-Avoidance Responsive Genes Are Dawn Specific

**Figure 7:**
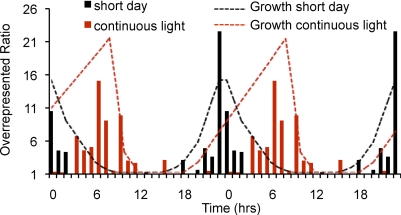
Shade-Avoidance Genes Overrepresented during the Growth Phase Genes misregulated using shade avoidance–like conditions are expressed at dawn and dusk, specifically under short-day photocycles (black) and continuous light (red). Overrepresentation ratio is double plotted for visualization purposes. Shade-avoidance microarray data were reanalyzed from Sessa et al. 2005 [36]. The growth curves for short-day photocycles (black dotted lines) and continuous light (red dotted lines) are reproduced for frame of reference.

Shade avoidance includes a plant's response to external lighting conditions when a plant experiences a change in the red (R) to far-red (FR) ratio (R/FR). The shade-avoidance response is characterized by increased stem elongation and changes in leaf morphology in mature plants that are similar to the etiolated responses in embryonic stems and leaves [35]. Similar to hypocotyl elongation, shade avoidance is gated by the circadian clock with maximal gating expression of *PIL1* and growth response around dusk under continuous light [2], similar to the coordination of phytohormone transcripts under the same condition. To test whether there is a time-of-day signature in shade-responsive genes, we analyzed genes that are differentially expressed by 1 h of shade treatment (low R/FR) [36] using PHASER. Genes that are up-regulated by the low R/FR treatment (*p* < 0.01) are highly overrepresented around dawn (*p* < 0.0001) or dusk (*p* < 0.0001) under short-day and continuous conditions, respectively (Figure 7). Of the 62 low R/FR up-regulated genes, 16 are in our list of phytohormone genes, consistent with these genes playing a role in the plant's adjustment to shade conditions. These results suggest that phytohormone transcript coordination could be involved with stem growth (and, potentially, changes in leaf patterns) in mature plants.

### Altering the Time-of-Day Expression of BR Perception Affects Growth

**Figure 8:**
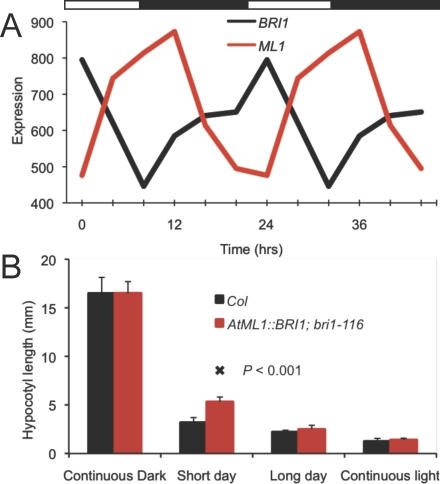
Time-of-Day–Specific Expression of BRI1 Is An Important Factor for Proper Hypocotyl Growth (A) The phase of *ML1* expression (black) is 12 h later than the phase of *BRI1* expression under short-day photocycles. Black bars above graph represent dark period of the day. (B) Proper photoperiodic hypocotyl elongation requires correct phasing of the BR perception. *AtML1::BRI1*, *bri1-116* (red bar) has longer hypocotyls under short-day photoperiods [37], compared to Col (black bar); *p* < 0.001, Student *t*-test. Plants were grown for 7 d under four different light conditions: continuous light, short days (8-h light/16-h dark), long days (16-h light/8-h dark), and continuous dark. All data represent the results from two independent experiments.

To establish whether the regulation of phytohormone transcript abundance during the growth phase of the day affects growth, we made use of recently described lines in which the BR receptor (BRASSINOSTEROID INSENSITIVE 1; BRI1) is expressed using the Arabidopsis thaliana meristem layer 1 (*AtML1*) promoter in a strong *bri1* mutant background, *AtML1::BRI1; bri1-116* [37]. These lines, which contain an equivalent amount of BRI1 protein as the wild type [37], were shown to rescue the severe growth defects of *bri1-116* under long-day photocycles (16-h light/ 8-h dark). Whereas the peak transcript abundance of both *AtML1* and *BRI1* transcripts occurs at dawn under long-day photocycles, consistent with rescue of *bri1-116*, under short-day photocycles, the peak of *AtML1* transcript abundance shifts to midday, 12 h later than *BRI1* (Figure 8A). Whereas under normal conditions *BRI* transcript abundance starts to increase in the dark with peak abundance near dawn, *AtML1::BRI1* expression is highest during the beginning of the dark period. We reasoned that shifting the peak of *BRI1* transcript abundance to the early evening with the *AtML1* promoter should result in longer hypocotyls under short days due to the increased overlap between *BRI* transcript abundance and the dark period. Consistent with our hypothesis, we found that *AtML1::BRI1; bri1-116* hypocotyls were slightly, but significantly, longer than wild type under short days (Figure 8B; *p* < 0.001). Hypocotyl length was the same as wild type under continuous light or dark, and long days as previously reported (Figure 8B) [37]. This result demonstrates that the timing of growth-associated phytohormone expression is important for wild-type growth. Furthermore, it suggests that *BRI1* expression reflects BRI1 protein activity, and that more BRI1 activity during the dark increases hypocotyl growth. The modest increase in hypocotyl length may reflect the fact that there is only a limited set of components in the phytohormone growth pathway available at that time of day, suggesting that the internal coincidence with other phytohormones is also important for growth.

## Discussion

**Figure 9:**
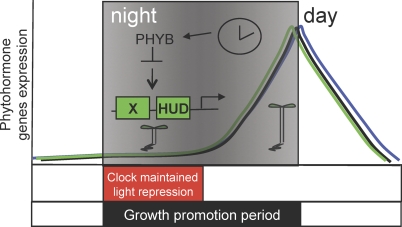
Model Describing Circadian-, Light-, and Phytohormone-Mediated Hypocotyl Growth The circadian clock and light signaling interact to coordinate a group of different phytohormone transcripts (represented by green, blue, and black lines) to coincide with the dark-to-light transition at dawn. Hypocotyl elongation is a mode of dark growth, so the dark phase of each day is the “growth promotion” period (black box). The circadian clock ensures that growth does not proceed immediately upon exposure to darkness by gating light signaling during the “circadian-maintained light repression” period (red box). The circadian clock maintains light repression during the early evening through light signaling and PHYB activity. In turn, PHYB activity acts indirectly through an unknown gene on at least two *cis*-acting elements, the HUD and an unknown X element, to repress phytohormone transcript abundance. As night proceeds, circadian maintenance of light signaling decreases, repression of phytohormone transcript abundance is released, and maximal growth occurs at dawn [15,17]. This model does not attempt to describe hormone abundance or activity. It specifically describes the coordination of phytohormone gene transcript abundance by the circadian clock and light signaling to coincide with the hypocotyl growth window. However, the predictive nature of this model provides a framework for future studies that will directly interrogate the specific interactions of hormones in controlling growth.

We have described a transcriptional module that is closely associated with increase in growth rate. It appears to be regulated by coordination of the internal coincidence of multiple phytohormone pathways with the external coincidence of a key environmental signal, the dark-to-light transition at dawn (Figure 9). Our analysis suggests that the circadian clock acts upstream of light signaling to gate phytohormone gene expression during the early evening. This program constitutes a key mechanism to ensure effective growth during the long periods of darkness early in *Arabidopsis* development (short days of fall or early spring) or low-light conditions experienced during shading. We demonstrate that PHYB (or light signaling) directly controls phytohormone transcript abundance and that this correlates with increased hypocotyl growth rate in *Arabidopsis*.

We propose a gated convergence model, to provide a framework to understand the intimate connection between the circadian clock, light signaling, and phytohormone control of growth that has eluded the field to date (Figure 9). In this model, the circadian clock mitigates the effect of darkness on growth by gating the expression of the growth-associated HUD-containing phytohormone genes. Although the molecular mechanism by which the circadian clock gates light signaling is yet to be identified, PHYB is downstream and directly controls the expression of phytohormone genes. At least two *cis*-acting elements, HUD and an unknown element, constitute the downstream targets of light signaling (and PHYB activation). As the night proceeds, circadian-mediated maintenance of light signaling diminishes and phytohormone transcript abundance increases, leading to maximal rate of growth at dawn. This model predicts that environment-tuned growth is governed by both the internal coincidence of phytohormone transcript abundance and external coincidence with the dark period of the diurnal cycle. Consistent with this model, when we misexpressed BRI1 to the early evening using the *ML1* promoter, we observed longer hypocotyls. The model predicts that an increase of the phytohormone genes during the dark phase of the day would lead to an increase in growth. This is what we observe in the circadian mutants, a *phyB* mutant and an *AtML1::BRI1* line grown under short-day photocycles.

The gated convergence model can be used to explain the transition from etiolated (dark) to de-etiolated (light) growth. In its typical geographic range, *Arabidopsis* germinates under natural conditions in late fall or early spring, both of which are short-day conditions. While the seedling is under the soil, it elongates rapidly and most likely continuously [15] until it breaks the soil and experiences its first light, which marks the beginning of the developmental shift to photomorphogenesis. Over the next 7 d, daily hypocotyl elongation will decrease and cotyledons will start to green and grow. However, the plant is still experiencing large segments (>12 h) of darkness, and must mitigate the effect of darkness on promoting growth. According to the gated convergence model, the circadian clock reads out day length and modulates the waveforms of cyclic phytohormone transcript abundance during the early night to slow hypocotyl growth. This model would also predict that the circadian clock provides some level of memory of developmental state, such as it does for flowering time [38]. Recently, it has been shown in multiple organisms, including *Arabidopsis*, that the circadian clock mediates changes in chromatin modifications [39–41], suggesting that these developmental states may be reflected in chromatin modifications that change transcriptional activities.

The gated convergence model provides a time-of-day framework for understanding the relationship between the circadian clock, light signaling, and phytohormone-regulated growth. One possible mechanism by which the circadian clock could mitigate increased growth and gene expression in the dark is by maintaining light signaling during the early evening through retention of the active form of PHYB in the nucleus. PHYB:GFP is imported into the nucleus in a light-dependent diurnal pattern [42]. Therefore, it is interesting to speculate that the circadian clock may also play a role in this process to maintain active light signaling in the early part of the night. Active forms of PHYB destabilize members of the PIF bHLH transcription factor family [43], which bind Gbox binding sites, like the HUD, to promote hypocotyl elongation [15]. In addition, the DELLA GA-signaling repressors bind PIF3 and PIF4 in the absence of GA, whereas they are degraded in the presence of GA, leading to the regulation of growth-promoting gene expression [43–45]. Therefore, the temporal coordination and balance of GA and light at dawn directly controls downstream growth-promoting pathways. Although some of these activities are at the level of protein activity, underlying transcriptional regulation of the PIFs [15] and other phytohormone signaling components provides a temporal window for this regulation. For instance, the circadian transcriptional regulation of the IAA-signaling pathway plays a pivotal role in how IAA-mediated growth proceeds [14].

In a previous study, we developed a time-of-day expression atlas and showed that photocycles, thermocycles, and the circadian clock control the transcript abundance of 90% of *Arabidopsis* genes [19]. The implication of this finding is that many pathways are coordinated and have maximum abundance at specific times over the day. Our results support that transcriptional coordination of phytohormone genes is indeed important for establishing temporal interactions of growth pathways. It is becoming increasingly clear that a unifying principle for circadian function in both plants and animals is the gating of convergent, stochastic signals such that physiological processes with complex inputs are provided a temporal organization. The temporal coordination of flux through systemic signaling pathways thus appears to be a universal feature of clock regulation from growth control in plants to blood pressure regulation in humans [46,47].

## Materials and Methods

### Plant genotypes and growth conditions.

All 2-d (12 time point) time courses were described [19]. The short-day time course was in the L*er* background. Plants were grown under short-day photocycles, 8-h light (180 μE/m^2^s)/16-h dark. The continuous-light time course utilized in this study was in the Columbia (Col) background. The seedlings for the continuous-light time course were grown under 12-h light (100 μE/m^2^s)/12-h dark for 7 d and then collected under continuous light and temperature over 2 d (12 time points). The *phyB-9* [32] and *lux-2* [30] mutants were in the Col background, and *lhy* [31] was in the L*er* background. *phyB-9* and *lhy* were grown under short-day conditions of 8-h light/16-h dark at 22 °C for 7 d and collected every 4 h over 1 d (six time points). The *lux-2* plants were grown under intermediate-day conditions of 12-h light/12-hs dark at 22 °C, and collected every 4 h over 1 d (six time points).

### Phytohormone gene list.

The phytohormone gene list was assembled from the literature based on genetic or expression data implicating them in the biosynthesis, catabolism, signaling, or reception of phytohormones.

### Phase overrepresentation plots (PHASER).

Phase overrepresentation was calculated as the number of genes with a given phase in a list, divided by the number of genes in that list, over the number of genes called rhythmic, divided by the total number of genes on the array. (Number of genes with phase X in a list/total number of genes in the list)/(Number of genes with phase X across on the array/total number of genes on the array). Phase overrepresentation is double plotted (one day of data plotted a second day) for visualization purposes. A Web-based implementation of phase overrepresentation plots called PHASER can be found at: http://phaser.cgrb.oregonstate.edu/.

### Microarray data and analysis.

All nonmutant microarray time courses were performed as described [19,22]. These time courses are available through our Web interface at DIURNAL: http://diurnal.cgrb.oregonstate.edu, DACARI: http://dacari.rutgers.edu/dacari/, and at ArrayExpress: E-MEXP-1304. Mutant microarray time course experiments were performed in the same manner. Briefly, tissue was collected and frozen in liquid nitrogen, pulverized, and then RNA was extracted using RNAeasy with DNAase on column treatment (QIAGEN), and labeled and hybridized to Affymetrix ATH1 GeneChip per Affymetrix protocol. Resulting CEL files were checked for array quality using standard tools implemented in the Bioconductor packages simpleaffy and affyPLM, and microarrays were normalized together using gcRMA [48]. Present/absent calls were made using the Affymetrix MAS5 program (Affymetrix).

Cycling gene calls and phase estimates were described for the short-day and continuous-light time courses [19]. Briefly, HAYSTACK, a model-based pattern-matching algorithm, was used to identify transcripts that change abundance over the day according to the hypothesis that if genes cycle- they will have a specific pattern that we can predefine (http://haystack.cgrb.oregonstate.edu) [22]. Transcripts were called cycling if their *p-*value for correlation to a specific pattern was less than 0.05. Phase estimates (in hours from dawn) were based on the time of peak transcript abundance [19]. For the 1-d time courses described in this study (*phyB-9*, *lhy*, *lux-2*, and associated parental genotypes Col, L*er*, and Col, respectively), data were double plotted and a factor of noise (δ) was introduced in order to reduce autocorrelation. Cycling transcripts were called with a *p* < 0.05, greater than 20 unlogged gene-chip Robust Multiarray Averaging (gcRMA) expression, and greater than 1.5-fold change between minimum and maximum expression to control for false positives introduced from the noise factor (δ).

For mutant or condition comparisons, gcRMA normalized data were fit using a linear model in the R Bioconductor limma package with a *p* < 0.01 cutoff. Datasets were downloaded from the ArrayExpress or GEO Web site: AtGenExpress light treatments GSE5617 and tissue (7-d-old cotyledons, hypocotyls, and roots), E-TABM-17; shade avoidance (low R/FR), E-MEXP-443 [36]; *ckx1-ox*, E-MEXP-344; *DELLApenta* (*ga1-3 gai-t6 rga-t2 rgl1-1 rgl2-1*) mutant, E-MEXP-849 [24]; *arf6-2 arf8-3*, GSE2848 [25]; *ein5-1*, GPL198 [26]; *abi1-1*, GSE6151; *brx*, E-MEXP-635&7 [27]. Whole datasets were downloaded from the respective Web sites, and all CEL files from a given experiment were normalized together, regardless of whether all conditions in the experiment were used to determine genes disrupted in the indicated mutant. Resulting gene lists were evaluated for time-of-day signatures using PHASER.

### *Z*-score profile of the HUD *cis*-regulatory element.

*Z*-score profiles for all diurnal and circadian elements are described [19]. *Z*-score profiles represent the overrepresentation of a specified word (3–8 bp) in the promoter (500 bp) of all *Arabidopsis* genes on the ATH1 GeneChip for a given phase. Overrepresentation was determined using ELEMENT, which is an enumerative promoter searching algorithm (http://element.cgrb.oregonstate.edu) [19,22,49]. *Z*-score profiles are double plotted for visualization purposes.

### Phytohormone genes associated with the growth phase of the day.

Phytohormone genes associated with the growth phase of the day were identified using the HAYSTACK model matching algorithm [19]. Four growth-associated patterns were chosen from the 182 phytohormone genes that were used as the models to search for similar patterns. Two types of patterns were used: dawn-box and dawn-spike (Figure 2). The expression patterns of *TIR1* and *ERS2* were used for dawn-box, and *AC8* and *GAOX6* were used for dawn-spike models. The 182 phytohormone genes were searched using these four models, and 71 highly correlated genes (*p* < 0.01) were identified (Table S2). These 71 phytohormone genes were used for subsequent analysis.

### Quantitative Real Time PCR.

qRT-PCR was previously described [50]. Plants were grown under either thermocycles (12-h 22 °C/12-h 12 °C) and continuous light or short days for 7 d, RNA was isolated every 4 h, first-strand cDNA synthesis was carried out with 5 μg of RNA, and all qRT-PCR reactions were run on a BioRAD myIQ system using SYBRgreen. qRT-PCR time courses are double plotted for visualization purposes. Data presented represent the results of two independent experiments. Primers will be supplied upon request.

### Hypocotyl length assays.

Hypocotyl assays were performed as described [51]. For hypocotyl length measurements, roughly ten seeds were stratified on plates for 4 d at 4 °C in dark, and then transferred to specific growth conditions. Seven days later, plants were flattened and imaged on a flatbed scanner. Hypocotyl lengths were measured using NIH Image. Data presented represents the results of at least three independent experiments.

### Promoter luciferase assay.

The *3xHUD::LUC* construct was made by ligating two long oligos containing the HUD (CACATG) into a vector containing the −101/+4 fragment of the NOS minimal promoter and modified firefly luciferase (luc+). Plants transformed with the empty plasmids did not confer any cyclic pattern to luciferase (unpublished data). Plasmids were transformed into the Col-0 accession using the floral dip method [52]. Except where indicated, seedlings were grown on MS medium (Gibco BRL) with 0.8% agar and 3% sucrose. Seedlings of the T1 generation were selected on kanamycin and transferred to soil for bulking. T3 seedlings were grown under nonselecting conditions before imaging. Wild-type seedlings were identified after image collection and removed from the analysis. During the initial week of growth, seedlings were all grown under light/dark at continuous 22 °C (LDHH) conditions and then 2 or 3 d prior to imaging, transferred to the proper entrainment condition (short day or continuous light) on smaller plates without sucrose. Over the course of 5 d, images of seedlings were collected using a cooled charged-coupling device (CCD) camera for 25 min every 2.5 h using the Wasabi software (Hamamatsu Photonics) using the slice photon counting mode. The images were quantified using the MetaMorph software (Universal Imaging) and graphed using Microsoft Excel (Microsoft). For each condition tested, six seedlings from each independent T3 line were analyzed in triplicate. In order to compare all independent T3 lines, each time series was normalized based on the respective median value. The average of the six T3 seedlings was plotted for each treatment.

## Supporting Information

Figure S1 Core Circadian Clock Components Have Essentially Wild-Type Expression under Short-Day Photocycles in the *phyB-9* Mutant (Red) Compared to the Col Parent (Blue) (A) *LUX* and (B) *LHY*. Despite the long hypocotyl phenotype of the *phyB-9* mutant, the expression of core circadian clock genes is not affected. This is consistent with reports that the *phyB-9* mutant does not have severe clock defects in white light [53]. Time courses are double plotted for visualization purposes.
(18 KB PDF)

Figure S2 Core Circadian Clock Components Are Phased 12 h Later in the *lhy* Mutant (A) *TOC1* normally has peak expression at dusk (L*er*, black line), yet has peak expression around dawn in the *lhy* mutant (red line).
(B) *CCA1* normally has peak expression at dawn (L*er*, black line), yet has peak expression around dusk in the *lhy* mutant (red line). Plants were grown and sampled under short-day photoperiods. Time course is double plotted for visualization purposes.
(16 KB PDF)

Figure S3 The *lhy* Mutant Grown under Short-Day Photocycles Continues to Have Growth Defects and Looks Like a Dark-Grown Plant after 4 wk of Growth The *lhy* mutant has very long petioles and small leaves, consistent with a strong shade-avoidance growth response.
(29 KB PDF)

Table S1 Phytohormone Gene Time (h) of Peak Expression Genes were identified from the literature based on genetic and expression data that implicated them in biosynthesis, catabolism, reception, and signaling of the six phytohormone pathways. The time of peak transcript abundance in hours from dawn (phase), and *p*-value for correlation to growth-associated models are presented. When a cell is blank, this means that a phytohormone gene was not identified as cycling. MIPS ID is the unique *Arabidopsis* gene identification number; Affy ID, the unique Affymetrix probe set identification number.
horm, hormone; SD, short day.
(63 KB PDF)

Table S2 Phytohormone Genes That Match Growth-Associated Models (32 KB PDF)

Table S3 The 3–8mer Words Overrepresented in the 71 Phytohormone Gene Promoters (500 bp) (38 KB PDF)

## 

### Accession Numbers

The Affymetrix ATH1 GeneChip data for the light and circadian mutants *phyB-9*, *lhy*, and *lux-2* described in this paper have been deposited at ArrayExpress under accession number E-MEX-1299. The raw data and a Web interface are also available at DIURNAL: http://diurnal.cgrb.oregonstate.edu/ or DACARI: http://dacari.rutgers.edu/dacari/.

## Abbreviations

ABA: abscisic acid
ACC: ethylene
*AtML1*: Arabidopsis thaliana meristem layer 1
BR: brassinosteroid
BRI1: BRASSINOSTEROID INSENSITIVE 1
CK: cytokinin
Col: Columbia
GA: gibberellins
HUD: hormone up at dawn
IAA: auxin
L*er*: Landsberg *erecta*
LHY: late elongated hypocotyl
LUC: luciferase
LUX: lux arrhythmo
PhyB: phytochrome B
PIF: phytochrome interacting factor
qRT-PCR: quantitative real-time PCR
