# Supplementary material for: A Morning-Specific Phytohormone Gene Expression Program underlying Rhythmic Plant Growth
Source: PLoS Biol. 2008 Sep 16;6(9):e225. doi: 10.1371/journal.pbio.0060225 (PMC2535664; doi:10.1371/journal.pbio.0060225)

Michael et al. Figure S2

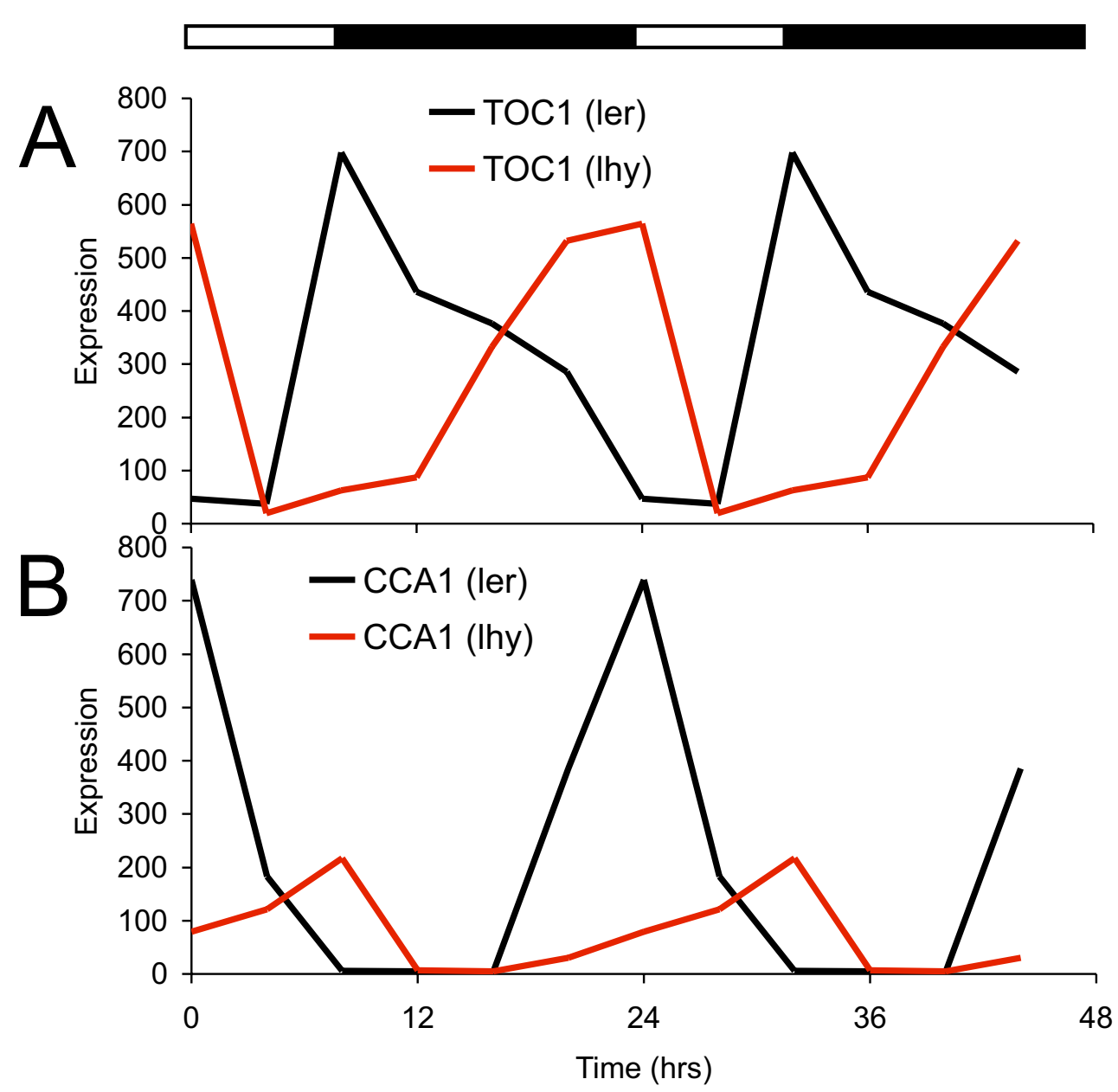

Supplement: Supplementary file 2 [file pbio.0060225.sg002.pdf]
