# Supplementary material for: A Morning-Specific Phytohormone Gene Expression Program underlying Rhythmic Plant Growth
Source: PLoS Biol. 2008 Sep 16;6(9):e225. doi: 10.1371/journal.pbio.0060225 (PMC2535664; doi:10.1371/journal.pbio.0060225)
Supplement: Supplementary file 4 [file pbio.0060225.st001.pdf]

Michael et al., Table S1. Phytohormone gene time (hrs) of peak expression

horm	name	func	Affy ID	AGI ID	SD	lhy	ler	phyb	col	lux	col
ABA	ABA3	biosynthesis	246325_At	At1g16540							
ABA	NCED3	biosynthesis	257280_At	At3g14440	3	19				6	4
ABA	ABA1	biosynthesis	247025_At	At5g67030	3	6	3	3	3	5	5
ABA	AAO3	biosynthesis	263570_At	At2g27150	4	2					
ABA	NCED2	biosynthesis	254668_At	At4g18350							
ABA	NCED9	biosynthesis	260797_At	At1g78390							
ABA	NCED5	biosynthesis	256190_At	At1g30100							
ABA	AAO2	biosynthesis	246330_At	At3g43600	5	6	6	6	5	8	8
ABA	NCED6	biosynthesis	257242_At	At3g24220	8						
ABA	ABA2	biosynthesis	259669_At	At1g52340	23	7		4		6	22
ABA	CYP707A1	catabolism	254562_At	At4g19230	0					5	6
ABA	CYP707A4	catabolism	257035_At	At3g19270	4						
ABA	CYP707A2	catabolism	266778_At	At2g29090	5					6	7
ABA	CYP707A3	catabolism	248964_At	At5g45340	23						
ABA	GUN5	receptor	250243_at	At5g13630	2	7	3	1	2	5	4
ABA	RPK1	receptor	260345_At	At1g69270	18	20	19	20	17	20	19
ABA	ABI4	signaling	263377_At	At2g40220							
ABA	ABI3	signaling	256898_At	At3g24650							
ABA	ABI2	signaling	247957_At	At5g57050	5	3		4		7	7
ABA	ABI1	signaling	253994_At	At4g26080	5	0	1	3		7	9
ABA	ANP2	signaling	256351_At	At1g54960							
ABA	ABI5	signaling	263907_At	At2g36270	7	21					
ABA	ARAC1	signaling	264593_At	At2g17800		6	6		7		
ABA	ANP1	signaling	264623_At	At1g09000							
ABA	SAD1	signaling	248678_At	At5g48870	14	10		11			20
ABA	ROP10	signaling	252375_At	At3g48040	23	17	23		18	18	17
ACC	ACS8	biosynthesis	253066_At	At4g37770	0		23		23	23	0
ACC	ACS4	biosynthesis	266830_At	At2g22810	0						
ACC	ACS3	biosynthesis	246172_s_At	At3g61510							
ACC	ACO	biosynthesis	249125_At	At5g43450	5			23	1	5	7
ACC	ACO	biosynthesis	249128_At	At5g43440	6			8	8	10	9
ACC	ETO3	biosynthesis	252279_At	At3g49700							
ACC	ACO1	biosynthesis	265948_At	At2g19590					7	4	
ACC	ACO	biosynthesis	247774_At	At5g58660	13	20	15		16		
ACC	ACS7	biosynthesis	253999_At	At4g26200	15		16				
ACC	ACS12	biosynthesis	248425_At	At5g51690	15						
ACC	ACO	biosynthesis	265615_At	At2g25450				7	7	9	9
ACC	EAT1	biosynthesis	265194_At	At1g05010	16	16	17	18	17	17	
ACC	ACO2	biosynthesis	260637_At	At1g62380	18		18	18	19	1	
ACC	ETO1	biosynthesis	246298_At	At3g51770	18	17	20		18	20	18
ACC	ACO	biosynthesis	264843_At	At1g03400	23	21	0	20	23	4	1
ACC	ACS2	biosynthesis	259439_At	At1g01480		0					
ACC	ACS6	biosynthesis	254926_At	At4g11280		3	4	4	4		
ACC	ETO2	biosynthesis	247159_At	At5g65800	23						
ACC	ETR2	receptor	257922_At	At3g23150	13	19	17	18	16		
ACC	ERS1	receptor	245098_At	At2g40940	13	18	17	17	16	22	17
ACC	EIN4	receptor	258818_At	At3g04580	16	10	16	16	14		17
ACC	ERS2	receptor	263653_At	At1g04310	18	21	17	19	17	23	

ACC	ETR1	receptor	260133_At	At1g66340	20	20					17
ACC	EIR1	signaling	247947_At	At5g57090		16		0		0	
ACC	CTR1	signaling	250911_At	At5g03730	13	19	17	17	17	20	15
ACC	EBF1	signaling	265633_At	At2g25490		18	18	17	17	21	15
ACC	EIN3	signaling	257981_At	At3g20770	18	19	18	20	18	22	17
ACC	EBF2	signaling	246935_At	At5g25350	18	18	18	19	17	22	18
ACC	EIN2	signaling	250928_At	At5g03280		20	19	21	18		18
ACC	HLS1	signaling	253054_At	At4g37580	18	18	19	21		23	
ACC	EIL1	signaling	266302_At	At2g27050	18	18	18	20	18	20	
BR	BRox2	biosynthesis	256598_At	At3g30180	0	3	22	22	2	23	1
BR	BRox1	biosynthesis	249484_At	At5g38970							
BR	DWF7	biosynthesis	258484_At	At3g02580	4	6		5	8		
BR	UgT73C5	biosynthesis	265200_s_At	At2g36790	6	5		4	5		
BR	DET2	biosynthesis	266095_At	At2g38050	8	18	15	17			17
BR	ROT3L	biosynthesis	256788_At	At3g13730	10						
BR	DWF5	biosynthesis	261865_At	At1g50430	18		12	13	9		
BR	CPD	biosynthesis	250752_At	At5g05690	18	20	18	22	22	21	20
BR	DWF1	biosynthesis	257938_At	At3g19820	20			21	18	22	20
BR	DWF4	biosynthesis	252184_At	At3g50660	22	1	20	23	16		14
BR	ROT3	biosynthesis	246216_At	At4g36380	23	22	0	22	1		
BR	SOB7	catabolism	262525_At	At1g17060	20	18	22	21		21	
BR	BAS1	catabolism	267614_At	At2g26710	23	18	23	23	0	18	23
BR	IMK2	receptor	252078_At	At3g51740	13						
BR	BRL3	receptor	256981_At	At3g13380	14	23	16			11	15
BR	BRL1	receptor	264537_At	At1g55610							
BR	BRL2	receptor	265250_At	At2g01950	18	6	19	23			20
BR	BRI1	receptor	252890_At	At4g39400	23		0			20	
BR	BEH4	signaling	264248_At	At1g78700	0	19	23	23	0	22	
BR	BIM1	signaling	250569_At	At5g08130	0	8	4	4		22	16
BR	BIM2	signaling	259645_At	At1g69010	0	23	1	23	0	23	23
BR	BEH1	signaling	252178_At	At3g50750	0	18	0	22	22	23	23
BR	BEH3	signaling	254610_At	At4g18890							20
BR	BAK1	signaling	253338_At	At4g33430	13	22			12		15
BR	SERK1	signaling	261521_at	At1g71830		7	4				16
BR	BSU1	signaling	264823_At	At1g03445							
BR	BKI1	signaling	249190_at	At5g42750	16	22	19	19	20	18	19
BR	BES1/BZR1	signaling	259955_s_At	At1g19350	17		18	17	18	19	19
BR	BEH2	signaling	246284_At	At4g36780	18	17	18	19	18	21	19
BR	BIN2	signaling	254616_At	At4g18710	18	19	18	20	18	19	20
BR	BRS1	signaling	253579_At	At4g30610	23	7	6	6	6		21
CK	AtIPT5	biosynthesis	249972_At	At5g19040							
CK	AtIPT4	biosynthesis	254129_At	At4g24650							
CK	AtIPT7	biosynthesis	258103_At	At3g23630							
CK	CYP735A1	biosynthesis	249566_At	At5g38450							
CK	AtIPT8	biosynthesis	257039_At	At3g19160							
CK	AtIPT6	biosynthesis	255741_At	At1g25410							
CK	AtIPT3	biosynthesis	251154_At	At3g63110		8					15
CK	AtIPT9	biosynthesis	246152_At	At5g20040	14	11	12	10		15	19
CK	UgT76C2	biosynthesis	250753_At	At5g05860	17		16	14	17	18	20

CK	UgT76C1	biosynthesis	250750_At	At5g05870	19	21					22
CK	CYP735A2	biosynthesis	264470_At	At1g67110	20						
CK	AtIPT1	biosynthesis	260240_At	At1g68460							
CK	CKX5	catabolism	261109_At	At1g75450	0	13		0	23	0	
CK	CKX1	catabolism	245108_At	At2g41510	2						
CK	CKX3	catabolism	247956_At	At5g56970	14						
CK	CKX2	catabolism	265945_At	At2g19500							
CK	CKX6	catabolism	251178_At	At3g63440							
CK	CKX4	catabolism	253696_At	At4g29740	17				5		
CK	AHK4	receptor	263599_At	At2g01830	15	7					16
CK	AHK2	receptor	249693_At	At5g35750	19	11	15	20		11	
CK	AHK3	receptor	264448_At	At1g27320		23	21	21	21		21
CK	CKI1	signaling	245125_At	At2g47430							
CK	ARR4	signalling	263236_At	At1g10470	14	9	11	7	12	12	16
CK	ARR7	signalling	259466_At	At1g19050	15	8		9		12	17
CK	ARR15	signalling	262212_At	At1g74890	15						
CK	ARR2	signalling	252374_At	At3g48100	17	16		0		7	18
CK	ARR8	signalling	266372_At	At2g41310	17	7	5		23		
CK	ARR16	signalling	266078_At	At2g40670	17		16		18		19
CK	ARR6	signalling	247406_At	At5g62920		8	8	7		10	17
CK	ARR9	signalling	251665_At	At3g57040	22	9	0	0	23	12	22
CK	PUP1	transport	245671_At	At1g28230	7	23	5	9	7	9	10
GA	ATGA3OX3	biosynthesis	254397_At	At4g21690							
GA	ATGA20OX5	biosynthesis	259453_At	At1g44090							
GA	GA1	biosynthesis	255461_At	At4g02780							
GA	ATGA20OX2	biosynthesis	248371_At	At5g51810	0	19					23
GA	AtKAO2	biosynthesis	266335_At	At2g32440		18	23	17			7
GA	GA5	biosynthesis	254065_At	At4g25420							
GA	GA4	biosynthesis	261768_At	At1g15550	2	9		4	4	23	3
GA	ADC2	biosynthesis	253203_at	At4g34710	2	5	18	16	18		21
GA	ATGA20OX3	biosynthesis	250611_At	At5g07200							
GA	GA4H	biosynthesis	260300_At	At1g80340	6						
GA	ATGA20OX	biosynthesis	248443_At	At5g51310	6						
GA	ATGA20OX4	biosynthesis	259714_At	At1g60980							
GA	ATGA3OX4	biosynthesis	260299_At	At1g80330							
GA	GA2	biosynthesis	262891_At	At1g79460	9				8	9	10
GA	ATGA20OX	biosynthesis	252530_At	At3g46500		19					
GA	AtKAO1	biosynthesis	264586_At	At1g05160		19	19	22	19		
GA	ADC1	biosynthesis	263241_at	At2g16500		6	5	7	0		
GA	GA3	biosynthesis	246864_At	At5g25900	22	16	18	0	18	23	21
GA	ATGA2OX6	catabolism	259445_At	At1g02400	0	22	17	21		21	
GA	ATGA2OX1	catabolism	260773_At	At1g78440	4					5	
GA	ATGA2OX7	catabolism	256211_At	At1g50960						5	
GA	ATGA2OX8	catabolism	254459_At	At4g21200	8						
GA	ATGA2OX4	catabolism	259614_At	At1g47990							
GA	ATGA2OX2	catabolism	260023_At	At1g30040	18						
GA	ATGA2OX3	catabolism	266995_At	At2g34500	23	0		0		0	23
GA	RGL2	homeostasis	259042_At	At3g03450	5	7					
GA	RGL1	homeostasis	260141_At	At1g66350	20		16		16	23	

GA	RGA1	homeostasis	266331_At	At2g01570	23	17				1	4
GA	GAI	homeostasis	262850_At	At1g14920	23	18	22	22		21	23
GA	RGL3	homeostasis	246432_At	At5g17490	23						
GA	ATGID1C	receptor	246782_At	At5g27320		18	19	20	12		
GA	ATGID1B	receptor	251200_At	At3g63010		22	16	19			19
GA	ATGID1A	receptor	259302_At	At3g05120	13	20	16		15		14
IAA	YUCCA-like	biosynthesis	253794_At	At4g28720	24	18	23	23	23	23	0
IAA	CYP79B2	biosynthesis	252827_At	At4g39950	4	6	6	6	13	8	
IAA	TRP2	biosynthesis	251847_At	At3g54640		6	10	9	10	11	
IAA	YUCCA6	biosynthesis	246900_At	At5g25620							
IAA	YUCCA1	biosynthesis	253439_At	At4g32540							
IAA	FASS	biosynthesis	249998_At	At5g18580	18			18	18		
IAA	AAO1	biosynthesis	246133_At	At5g20960						13	
IAA	YUCCA2	biosynthesis	254758_At	At4g13260	23	13					
IAA	SUR2	catabolism	253534_At	At4g31500	4	7	9	10	11	11	14
IAA	SUR1	catabolism	263714_At	At2g20610	6	6	7	6	7	9	8
IAA	IAMT1	conjugAtion	248104_At	At5g55250	0						
IAA	gH3.5	conjugAtion	253908_At	At4g27260	24	18	23	23	23	21	23
IAA	DFL2	conjugAtion	255403_At	At4g03400	5	8	3	5		6	7
IAA	ILR1	conjugAtion	258610_At	At3g02875	19	22					23
IAA	IAR3	conjugAtion	256178_s_At	At1g51760	20		18		16	12	
IAA	gH3.6	conjugAtion	248163_At	At5g54510	23	18	21	19	20	18	20
IAA	ACX1	IBA metabolism	245249_At	At4g16760	3	22	21	22	22	3	3
IAA	PED2	IBA metabolism	247422_at	At5g62810	5	5			4	11	14
IAA	PEX6	IBA metabolism	263170_at	At1g03000	5	16				15	17
IAA	ACX4	IBA metabolism	246304_At	At3g51840	18	20	18	20	22	21	20
IAA	TIR1	receptor	251199_At	At3g62980	18	19	20	23	23	19	19
IAA	IAA14	signaling	245593_At	At4g14550	0		1	23	2		1
IAA	IAA29	signaling	253423_At	At4g32280	24	17	22		23	21	23
IAA	ARF18	signaling	251289_At	At3g61830	1	14	0	23	1	23	
IAA	IAA7	signaling	257769_At	At3g23050	18	20		18	18	23	
IAA	IAA3	signaling	263656_At	At1g04240	21	18	23	19	4	20	
IAA	IAA19	signaling	258399_At	At3g15540	23	18	1		1	23	0
IAA	IAA1	signaling	245397_At	At4g14560	23	20	22			0	4
IAA	IAA2	signaling	257766_At	At3g23030	23	19	20	20	22	20	23
IAA	IAA6	signaling	260152_At	At1g52830	23					23	23

*Numbers represent time of peak transcript abudance in hours after dawn or subjective dawn (circadian conditions).

horm, hormone; SD, short day photocycles; Col, Columbia; Ler, Landsberg-erecta

lhy, late elongated hypocotyl; lux, lux arrhythmo; b9, phyB-9; DD, continuous dark; LL, continuous light

Affy ID, Affymetrix ATH1 Genechip probe identification number; MIPS ID, Arabidopsis thaliana gene identification number

GA, Gibberellins; IAA, auxin; BR, brassinosteroid; ABA, abscisic acid; CK, cytokinin; ACC, ethylene
