# Supplementary material for: A Morning-Specific Phytohormone Gene Expression Program underlying Rhythmic Plant Growth
Source: PLoS Biol. 2008 Sep 16;6(9):e225. doi: 10.1371/journal.pbio.0060225 (PMC2535664; doi:10.1371/journal.pbio.0060225)
Supplement: Supplementary file 5 [file pbio.0060225.st002.pdf]

Table S2. Phytohormone genes that match growth-associated models

Affy ID	Ler	p	lhy	p	Ler (lhy)	p	lux-2	p	phyb	p	col	p
245098_at	dawn-box_ERS2	0.02	dawn-box_ERS2	0.00	dawn-box_ERS2	0.000	dawn-spike_GAOX6	0.00	dawn-box_ERS2	0.00	dawn-box_ERS2	0.00
245108_at	dawn-spike_GAOX6	0.01	dawn-box_ERS2	0.02	dawn-spike_GAOX6	0.370	dawn-spike_AC8	0.00	dawn-box_ERS2	0.30	dawn-spike_AC8	0.29
245125_at	dawn-spike_AC8	0.82	dawn-spike_AC8	0.31	dawn-spike_AC8	0.024	dawn-spike_GAOX6	0.00	dawn-box_ERS2	0.15	dawn-spike_AC8	0.78
245249_at	dawn-spike_GAOX6	0.38	dawn-spike_GAOX6	0.01	dark-box_TIR1	0.000	dawn-spike_AC8	0.08	dark-box_TIR1	0.00	dawn-spike_GAOX6	0.00
245397_at	dawn-spike_AC8	0.00	dark-box_TIR1	0.00	dawn-spike_GAOX6	0.000	dark-box_TIR1	0.32	dark-box_TIR1	0.18	dawn-spike_AC8	0.32
245593_at	*dawn-spike_GAOX6*	*0.00*	*dawn-box_ERS2*	*0.57*	*dawn-spike_GAOX6*	*0.007*	*dawn-spike_GAOX6*	*0.02*	*dawn-spike_GAOX6*	*0.00*	*dawn-spike_GAOX6*	*0.01*
245671_at	dawn-spike_AC8	0.84	dawn-spike_GAOX6	0.00	dawn-spike_AC8	0.886	dawn-spike_AC8	0.92	dawn-spike_AC8	0.57	dawn-spike_AC8	0.79
246133_at	dawn-box_ERS2	0.22	dawn-spike_GAOX6	0.64	dawn-box_ERS2	0.064	dawn-box_ERS2	0.22	dark-box_TIR1	0.04	dawn-spike_AC8	0.00
246152_at	dawn-box_ERS2	0.17	dawn-box_ERS2	0.60	dawn-box_ERS2	0.507	dawn-box_ERS2	0.02	dawn-box_ERS2	0.72	dawn-box_ERS2	0.03
246172_s_at	dawn-spike_GAOX6	0.08	dawn-box_ERS2	0.67	dawn-spike_AC8	0.100	dawn-spike_AC8	0.04	dawn-box_ERS2	0.13	dark-box_TIR1	0.03
246216_at	dawn-spike_GAOX6	0.00	dark-box_TIR1	0.01	dawn-spike_GAOX6	0.000	dawn-spike_GAOX6	0.16	dark-box_TIR1	0.00	dawn-spike_GAOX6	0.02
246284_at	dark-box_TIR1	0.02	dawn-box_ERS2	0.00	dawn-box_ERS2	0.000	dark-box_TIR1	0.00	dawn-box_ERS2	0.00	dawn-box_ERS2	0.00
246298_at	dark-box_TIR1	0.02	dawn-box_ERS2	0.00	dark-box_TIR1	0.000	dawn-box_ERS2	0.02	dawn-spike_AC8	0.00	dark-box_TIR1	0.01
246304_at	dawn-box_ERS2	0.00	dark-box_TIR1	0.00	dawn-box_ERS2	0.000	dark-box_TIR1	0.02	dark-box_TIR1	0.00	dawn-spike_GAOX6	0.00
246325_at	dawn-spike_GAOX6	0.03	dawn-spike_GAOX6	0.00	dawn-box_ERS2	0.522	dawn-spike_AC8	0.81	dawn-spike_GAOX6	0.00	dawn-box_ERS2	0.03
246330_at	dawn-spike_AC8	0.89	dawn-spike_AC8	0.94	dawn-spike_AC8	0.870	dawn-spike_AC8	0.97	dawn-spike_AC8	0.81	dark-box_TIR1	0.93
246432_at	dawn-spike_AC8	0.00	dark-box_TIR1	0.00	dawn-box_ERS2	0.002	dawn-spike_GAOX6	0.00	dark-box_TIR1	0.00	dark-box_TIR1	0.00
246782_at	dawn-spike_AC8	0.75	dark-box_TIR1	0.00	dawn-box_ERS2	0.000	dawn-box_ERS2	0.03	dark-box_TIR1	0.00	dawn-box_ERS2	0.02
246864_at	dark-box_TIR1	0.00	dawn-box_ERS2	0.00	dawn-box_ERS2	0.000	dawn-spike_GAOX6	0.10	dawn-spike_AC8	0.00	dawn-spike_GAOX6	0.00
246900_at	dawn-box_ERS2	0.13	dawn-spike_AC8	0.97	dawn-spike_AC8	0.982	dark-box_TIR1	0.02	dawn-spike_AC8	0.04	dawn-box_ERS2	0.50
246935_at	dawn-box_ERS2	0.04	dawn-box_ERS2	0.00	dawn-box_ERS2	0.000	dawn-spike_GAOX6	0.00	dawn-box_ERS2	0.00	dawn-box_ERS2	0.00
247025_at	dawn-spike_AC8	0.36	dawn-spike_AC8	0.90	dawn-spike_GAOX6	0.258	dawn-spike_AC8	0.78	dawn-spike_GAOX6	0.29	dawn-spike_GAOX6	0.26
247159_at	dawn-spike_AC8	0.00	dawn-box_ERS2	0.00	dawn-spike_AC8	0.024	dawn-spike_GAOX6	0.00	dawn-spike_AC8	0.48	dawn-spike_AC8	0.00
247406_at	dark-box_TIR1	0.22	dawn-spike_AC8	0.39	dawn-spike_AC8	0.344	dawn-box_ERS2	0.92	dawn-spike_AC8	0.63	dawn-spike_AC8	0.00
247422_at	dawn-box_ERS2	0.95	dawn-spike_GAOX6	0.52	dawn-spike_AC8	0.808	dawn-box_ERS2	0.33	dawn-spike_GAOX6	0.90	dark-box_TIR1	0.75
247774_at	dawn-box_ERS2	0.02	dark-box_TIR1	0.00	dawn-box_ERS2	0.008	dawn-box_ERS2	0.03	dark-box_TIR1	0.00	dawn-box_ERS2	0.00
247947_at	dawn-box_ERS2	0.23	dark-box_TIR1	0.03	dawn-spike_AC8	0.589	dawn-spike_GAOX6	0.00	dawn-spike_AC8	0.00	dawn-spike_AC8	0.69
247956_at	dawn-box_ERS2	0.46	dawn-box_ERS2	0.90	dawn-spike_AC8	0.393	dawn-spike_GAOX6	0.00	dark-box_TIR1	0.22	dawn-spike_AC8	0.69
247957_at	dawn-spike_GAOX6	0.61	dawn-spike_GAOX6	0.29	dawn-spike_GAOX6	0.292	dawn-spike_AC8	0.80	dawn-spike_GAOX6	0.66	dawn-spike_AC8	0.71
248104_at	dawn-spike_GAOX6	0.00	dawn-spike_GAOX6	0.00	dawn-spike_AC8	0.031	dark-box_TIR1	0.00	dawn-box_ERS2	0.00	dark-box_TIR1	0.54
248163_at	dawn-spike_AC8	0.00	dawn-box_ERS2	0.00	dark-box_TIR1	0.000	dark-box_TIR1	0.05	dawn-box_ERS2	0.00	dark-box_TIR1	0.00
248371_at	dawn-spike_AC8	0.00	dark-box_TIR1	0.00	dawn-spike_AC8	0.000	dark-box_TIR1	0.24	dawn-spike_AC8	0.00	dawn-spike_AC8	0.07
248425_at	dawn-box_ERS2	0.09	dawn-box_ERS2	0.00	dawn-box_ERS2	0.000	dawn-box_ERS2	0.00	dark-box_TIR1	0.00	dawn-box_ERS2	0.00
248443_at	dawn-spike_GAOX6	0.74	dawn-box_ERS2	0.97	dark-box_TIR1	0.914	dawn-spike_AC8	0.97	dawn-box_ERS2	0.84	dawn-spike_AC8	0.87
248678_at	dawn-box_ERS2	0.03	dawn-box_ERS2	0.98	dawn-spike_AC8	0.940	dark-box_TIR1	0.10	dawn-box_ERS2	0.69	dawn-spike_AC8	0.68

Table S2. Phytohormone genes that match growth-associated models

248964_at	dawn-spike_GAOX6	0.00	dawn-spike_AC8	0.00	dawn-spike_GAOX6	0.000	dawn-spike_AC8	0.86	dawn-spike_AC8	0.05	dawn-spike_GAOX6	0.42
249125_at	dawn-spike_AC8	0.67	dawn-box_ERS2	0.72	dawn-spike_GAOX6	0.396	dawn-spike_AC8	0.75	dawn-spike_GAOX6	0.02	dawn-spike_GAOX6	0.08
249128_at	dawn-spike_AC8	0.87	dawn-box_ERS2	0.80	dawn-box_ERS2	0.568	dawn-box_ERS2	0.90	dawn-spike_AC8	0.70	dawn-spike_AC8	0.81
249190_at	dawn-box_ERS2	0.00	dawn-spike_GAOX6	0.00	dawn-box_ERS2	0.006	dawn-box_ERS2	0.00	dawn-box_ERS2	0.00	dark-box_TIR1	0.00
249484_at	dawn-spike_AC8	0.30	dawn-spike_GAOX6	0.13	dark-box_TIR1	0.785	dawn-spike_GAOX6	0.42	dawn-box_ERS2	0.01	dawn-spike_GAOX6	0.00
249566_at	dawn-spike_AC8	0.17	dawn-box_ERS2	0.28	dawn-box_ERS2	0.281	dawn-spike_AC8	0.00	dawn-spike_AC8	0.71	dawn-box_ERS2	0.73
249693_at	dawn-box_ERS2	0.02	dawn-box_ERS2	0.65	dawn-box_ERS2	0.023	dawn-box_ERS2	0.38	dark-box_TIR1	0.01	dawn-box_ERS2	0.64
249972_at	dawn-spike_GAOX6	0.01	dawn-box_ERS2	0.04	dawn-box_ERS2	0.826	dawn-spike_AC8	0.04	dark-box_TIR1	0.00	dawn-spike_AC8	0.20
249998_at	dark-box_TIR1	0.00	dawn-box_ERS2	0.00	dark-box_TIR1	0.000	dark-box_TIR1	0.00	dawn-spike_GAOX6	0.00	dawn-box_ERS2	0.00
250243_at	dawn-spike_AC8	0.09	dawn-spike_AC8	0.91	dawn-spike_AC8	0.085	dawn-spike_AC8	0.61	dawn-spike_GAOX6	0.21	dawn-spike_GAOX6	0.07
250569_at	dawn-spike_AC8	0.00	dawn-spike_AC8	0.82	dawn-spike_AC8	0.046	dawn-spike_GAOX6	0.00	dawn-spike_AC8	0.23	dawn-spike_AC8	0.02
250611_at	dawn-spike_AC8	0.21	dawn-spike_AC8	0.00	dark-box_TIR1	0.005	dark-box_TIR1	0.00	dark-box_TIR1	0.63	dawn-spike_AC8	0.01
250750_at	dark-box_TIR1	0.00	dark-box_TIR1	0.01	dawn-box_ERS2	0.004	dawn-box_ERS2	0.89	dark-box_TIR1	0.03	dawn-spike_GAOX6	0.03
250752_at	dark-box_TIR1	0.00	dark-box_TIR1	0.00	dark-box_TIR1	0.001	dawn-spike_GAOX6	0.00	dawn-spike_GAOX6	0.00	dark-box_TIR1	0.00
250753_at	dawn-box_ERS2	0.00	dawn-box_ERS2	0.30	dawn-box_ERS2	0.000	dawn-box_ERS2	0.01	dawn-box_ERS2	0.05	dawn-box_ERS2	0.00
250911_at	dawn-box_ERS2	0.05	dark-box_TIR1	0.00	dawn-box_ERS2	0.000	dark-box_TIR1	0.00	dawn-box_ERS2	0.00	dawn-box_ERS2	0.00
250928_at	dark-box_TIR1	0.05	dark-box_TIR1	0.00	dark-box_TIR1	0.000	dawn-spike_AC8	0.00	dark-box_TIR1	0.00	dawn-box_ERS2	0.00
251154_at	*dawn-box_ERS2*	*0.04*	*dawn-spike_AC8*	*0.73*	*dawn-spike_AC8*	*0.001*	*dark-box_TIR1*	*0.00*	*dawn-box_ERS2*	*0.13*	*dawn-box_ERS2*	*0.00*
251178_at	dawn-box_ERS2	0.05	dawn-box_ERS2	0.00	dark-box_TIR1	0.000	dark-box_TIR1	0.12	dawn-box_ERS2	0.30	dawn-box_ERS2	0.18
251199_at	dark-box_TIR1	0.00	dark-box_TIR1	0.00	dark-box_TIR1	0.000	dawn-box_ERS2	0.00	dawn-spike_AC8	0.00	dawn-spike_GAOX6	0.00
251200_at	dawn-spike_AC8	0.44	dawn-spike_GAOX6	0.00	dawn-box_ERS2	0.000	dawn-box_ERS2	0.56	dark-box_TIR1	0.00	dawn-spike_AC8	0.08
251289_at	dawn-spike_GAOX6	0.00	dawn-box_ERS2	0.04	dawn-spike_AC8	0.000	dawn-spike_GAOX6	0.00	dawn-spike_GAOX6	0.00	dawn-spike_AC8	0.00
251665_at	*dark-box_TIR1*	*0.00*	*dawn-spike_AC8*	*0.71*	*dawn-spike_GAOX6*	*0.000*	*dawn-box_ERS2*	*0.48*	*dawn-spike_GAOX6*	*0.05*	*dawn-spike_GAOX6*	*0.00*
251847_at	dawn-box_ERS2	0.71	dawn-spike_AC8	0.09	dawn-box_ERS2	0.909	dawn-box_ERS2	0.68	dawn-spike_AC8	0.93	dawn-box_ERS2	0.89
252078_at	dawn-box_ERS2	0.06	dawn-box_ERS2	0.06	dawn-box_ERS2	0.117	dawn-box_ERS2	0.03	dawn-box_ERS2	0.51	dawn-box_ERS2	0.46
252178_at	dawn-spike_GAOX6	0.00	dawn-box_ERS2	0.00	dark-box_TIR1	0.033	dawn-spike_GAOX6	0.00	dawn-spike_GAOX6	0.00	dark-box_TIR1	0.00
252184_at	dawn-spike_GAOX6	0.01	dawn-spike_AC8	0.00	dark-box_TIR1	0.006	dawn-box_ERS2	0.80	dawn-spike_AC8	0.00	dawn-spike_AC8	0.10
252279_at	dawn-box_ERS2	0.65	dawn-box_ERS2	0.22	dawn-spike_AC8	0.019	dawn-spike_GAOX6	0.01	dawn-spike_GAOX6	0.14	dawn-spike_GAOX6	0.19
252374_at	dawn-box_ERS2	0.00	dawn-spike_AC8	0.22	dawn-spike_AC8	0.006	dawn-spike_AC8	0.89	dawn-spike_AC8	0.00	dawn-spike_AC8	0.01
252375_at	dawn-spike_AC8	0.00	dawn-box_ERS2	0.00	dawn-spike_AC8	0.000	dawn-box_ERS2	0.00	dawn-spike_AC8	0.02	dark-box_TIR1	0.00
252530_at	dawn-box_ERS2	0.31	dawn-box_ERS2	0.00	dawn-box_ERS2	0.000	dawn-spike_GAOX6	0.00	dawn-spike_AC8	0.00	dawn-box_ERS2	0.02
252827_at	dawn-spike_GAOX6	0.67	dawn-spike_AC8	0.58	dawn-spike_AC8	0.913	dawn-box_ERS2	0.93	dawn-spike_AC8	0.98	dawn-box_ERS2	0.33
252890_at	dawn-spike_AC8	0.00	dawn-spike_GAOX6	0.00	dawn-spike_GAOX6	0.004	dark-box_TIR1	0.01	dawn-spike_GAOX6	0.13	dawn-spike_GAOX6	0.00
253054_at	dawn-box_ERS2	0.01	dawn-box_ERS2	0.00	dawn-box_ERS2	0.000	dawn-spike_GAOX6	0.00	dark-box_TIR1	0.00	dark-box_TIR1	0.00
253066_at	dawn-spike_AC8	0.00	dawn-spike_AC8	0.03	dawn-spike_AC8	0.000	dawn-spike_GAOX6	0.00	dawn-spike_GAOX6	0.02	dawn-spike_AC8	0.00
253203_at	*dawn-spike_AC8*	*0.10*	*dawn-spike_AC8*	*0.89*	*dawn-box_ERS2*	*0.000*	*dawn-spike_AC8*	*0.10*	*dawn-box_ERS2*	*0.00*	*dawn-box_ERS2*	*0.00*

Table S2. Phytohormone genes that match growth-associated models

253338_at	dawn-box_ERS2	0.25	dark-box_TIR1	0.00	dawn-spike_AC8	0.613	dawn-box_ERS2	0.02	dawn-box_ERS2	0.11	dawn-box_ERS2	0.07
253423_at	dawn-spike_AC8	0.00	dawn-box_ERS2	0.00	dawn-spike_GAOX6	0.000	dark-box_TIR1	0.00	dawn-spike_GAOX6	0.02	dawn-spike_GAOX6	0.00
253439_at	dawn-box_ERS2	0.15	dawn-spike_AC8	0.00	dark-box_TIR1	0.001	dawn-spike_AC8	0.00	dawn-spike_GAOX6	0.14	dawn-spike_AC8	0.00
253534_at	dawn-spike_GAOX6	0.77	dawn-spike_AC8	0.62	dawn-box_ERS2	0.971	dawn-box_ERS2	0.90	dawn-spike_AC8	0.97	dawn-box_ERS2	0.77
253579_at	dark-box_TIR1	0.06	dawn-spike_AC8	0.87	dawn-spike_AC8	0.850	dark-box_TIR1	0.55	dawn-spike_AC8	0.88	dawn-spike_AC8	0.88
253696_at	dark-box_TIR1	0.01	dawn-spike_AC8	0.63	dawn-spike_AC8	0.080	dawn-spike_AC8	0.12	dawn-spike_AC8	0.78	dawn-spike_AC8	0.50
253794_at	dawn-spike_AC8	0.00	dawn-box_ERS2	0.00	dawn-spike_GAOX6	0.000	dawn-spike_GAOX6	0.00	dawn-spike_GAOX6	0.00	dawn-spike_GAOX6	0.00
253908_at	dawn-spike_AC8	0.00	dark-box_TIR1	0.00	dawn-spike_GAOX6	0.000	dawn-spike_GAOX6	0.00	dawn-spike_GAOX6	0.00	dawn-spike_GAOX6	0.00
253994_at	dawn-spike_AC8	0.68	dawn-spike_GAOX6	0.00	dawn-spike_GAOX6	0.000	dawn-spike_AC8	0.73	dawn-spike_GAOX6	0.25	dawn-box_ERS2	0.90
253999_at	dawn-box_ERS2	0.31	dawn-spike_AC8	0.00	dark-box_TIR1	0.061	dawn-spike_GAOX6	0.19	dawn-box_ERS2	0.17	dawn-spike_AC8	0.21
254065_at	dawn-spike_AC8	0.01	dark-box_TIR1	0.00	dawn-spike_GAOX6	0.004	dawn-spike_AC8	0.26	dawn-spike_GAOX6	0.62	dawn-spike_AC8	0.01
254129_at	dawn-spike_AC8	0.47	dawn-spike_AC8	0.16	dawn-box_ERS2	0.007	dawn-spike_AC8	0.00	dawn-spike_AC8	0.06	dark-box_TIR1	0.69
254397_at	dark-box_TIR1	0.71	dawn-spike_AC8	0.15	dawn-box_ERS2	0.467	dawn-spike_AC8	0.00	dark-box_TIR1	0.00	dark-box_TIR1	0.55
254459_at	dawn-spike_AC8	0.49	dark-box_TIR1	0.00	dawn-box_ERS2	0.144	dawn-spike_AC8	0.00	dawn-box_ERS2	0.08	dawn-box_ERS2	0.00
254562_at	dawn-spike_GAOX6	0.00	dawn-spike_GAOX6	0.00	dawn-spike_AC8	0.000	dawn-spike_GAOX6	0.77	dawn-spike_AC8	0.23	dawn-spike_AC8	0.68
254610_at	dawn-spike_AC8	0.38	dawn-spike_AC8	0.23	dark-box_TIR1	0.052	dark-box_TIR1	0.22	dawn-spike_AC8	0.68	dawn-spike_AC8	0.34
254616_at	dark-box_TIR1	0.00	dawn-box_ERS2	0.00	dawn-box_ERS2	0.000	dawn-box_ERS2	0.00	dark-box_TIR1	0.00	dark-box_TIR1	0.00
254668_at	dawn-spike_AC8	0.08	dawn-box_ERS2	0.25	dawn-box_ERS2	0.818	dawn-spike_AC8	0.00	dawn-spike_AC8	0.23	dawn-spike_AC8	0.00
254758_at	dawn-spike_AC8	0.00	dawn-box_ERS2	0.02	dawn-spike_AC8	0.153	dawn-spike_AC8	0.00	dawn-box_ERS2	0.05	dawn-spike_AC8	0.00
254926_at	dawn-spike_AC8	0.00	dawn-spike_AC8	0.53	dawn-spike_GAOX6	0.634	dawn-box_ERS2	0.51	dawn-spike_GAOX6	0.68	dawn-spike_GAOX6	0.65
255403_at	dawn-spike_GAOX6	0.76	dawn-spike_AC8	0.57	dawn-spike_AC8	0.534	dawn-spike_AC8	0.90	dawn-spike_AC8	0.54	dawn-spike_AC8	0.87
255461_at	dawn-spike_AC8	0.21	dawn-spike_AC8	0.05	dawn-box_ERS2	0.267	dawn-spike_GAOX6	0.53	dawn-spike_AC8	0.13	dawn-spike_AC8	0.01
255741_at	dawn-spike_AC8	0.60	dawn-box_ERS2	0.01	dawn-spike_AC8	0.185	dawn-spike_GAOX6	0.00	dawn-spike_AC8	0.07	dawn-box_ERS2	0.89
256178_s_at	dark-box_TIR1	0.00	dawn-box_ERS2	0.03	dawn-box_ERS2	0.000	dawn-box_ERS2	0.69	dawn-spike_GAOX6	0.00	dawn-box_ERS2	0.00
256190_at	dawn-spike_GAOX6	0.35	dawn-spike_AC8	0.00	dawn-spike_AC8	0.004	dawn-spike_AC8	0.00	dawn-spike_GAOX6	0.25	dawn-spike_AC8	0.00
256211_at	dark-box_TIR1	0.66	dawn-box_ERS2	0.00	dawn-spike_AC8	0.002	dawn-spike_AC8	0.78	dark-box_TIR1	0.01	dawn-spike_GAOX6	0.47
256351_at	dawn-spike_AC8	0.22	dawn-spike_AC8	0.69	dawn-box_ERS2	0.864	dawn-spike_AC8	0.00	dawn-spike_AC8	0.55	dawn-spike_GAOX6	0.06
256598_at	dawn-spike_AC8	0.00	dawn-spike_GAOX6	0.14	dark-box_TIR1	0.000	dawn-spike_AC8	0.00	dawn-spike_GAOX6	0.00	dawn-spike_AC8	0.00
256788_at	dawn-box_ERS2	0.39	dawn-box_ERS2	0.01	dawn-box_ERS2	0.575	dawn-box_ERS2	0.16	dawn-spike_GAOX6	0.01	dawn-spike_AC8	0.83
256898_at	dawn-spike_AC8	0.03	dark-box_TIR1	0.21	dawn-spike_GAOX6	0.068	dawn-spike_GAOX6	0.00	dawn-box_ERS2	0.11	dark-box_TIR1	0.12
256981_at	dawn-box_ERS2	0.20	dawn-spike_GAOX6	0.00	dawn-box_ERS2	0.006	dawn-box_ERS2	0.45	dawn-spike_AC8	0.01	dawn-box_ERS2	0.66
257035_at	dawn-spike_GAOX6	0.69	dawn-spike_AC8	0.73	dawn-spike_AC8	0.162	dawn-spike_AC8	0.81	dawn-spike_GAOX6	0.65	dawn-spike_GAOX6	0.56
257039_at	dawn-spike_AC8	0.93	dawn-box_ERS2	0.63	dawn-box_ERS2	0.210	dawn-spike_AC8	0.00	dark-box_TIR1	0.22	dawn-spike_AC8	0.06
257242_at	dawn-spike_AC8	0.83	dawn-spike_AC8	0.16	dawn-spike_GAOX6	0.309	dawn-spike_AC8	0.01	dawn-spike_AC8	0.01	dawn-box_ERS2	0.14
257280_at	dawn-spike_GAOX6	0.30	dark-box_TIR1	0.30	dawn-spike_GAOX6	0.002	dawn-spike_AC8	0.54	dawn-spike_GAOX6	0.65	dawn-spike_GAOX6	0.63
257766_at	dawn-spike_GAOX6	0.00	dark-box_TIR1	0.00	dark-box_TIR1	0.000	dark-box_TIR1	0.00	dawn-box_ERS2	0.00	dark-box_TIR1	0.00

Table S2. Phytohormone genes that match growth-associated models

257769_at	dark-box_TIR1	0.01	dark-box_TIR1	0.00	dark-box_TIR1	0.000	dawn-spike_GAOX6	0.00	dawn-box_ERS2	0.00	dawn-box_ERS2	0.00
257922_at	dawn-box_ERS2	0.10	dark-box_TIR1	0.00	dawn-box_ERS2	0.000	dawn-spike_GAOX6	0.01	dawn-box_ERS2	0.00	dawn-box_ERS2	0.00
257938_at	dark-box_TIR1	0.00	dark-box_TIR1	0.26	dark-box_TIR1	0.012	dark-box_TIR1	0.00	dawn-spike_GAOX6	0.00	dawn-box_ERS2	0.00
257981_at	dark-box_TIR1	0.01	dark-box_TIR1	0.00	dark-box_TIR1	0.000	dark-box_TIR1	0.00	dark-box_TIR1	0.00	dawn-box_ERS2	0.00
258103_at	dawn-box_ERS2	0.73	dawn-spike_GAOX6	0.00	dawn-spike_AC8	0.004	dawn-spike_AC8	0.00	dawn-box_ERS2	0.63	dawn-spike_GAOX6	0.04
258399_at	dawn-spike_AC8	0.00	dawn-box_ERS2	0.00	dawn-spike_AC8	0.000	dawn-spike_GAOX6	0.00	dawn-spike_GAOX6	0.07	dawn-spike_AC8	0.01
258484_at	dawn-spike_AC8	0.31	dawn-spike_AC8	0.98	dawn-spike_AC8	0.464	dawn-spike_GAOX6	0.45	dawn-spike_AC8	0.41	dawn-spike_AC8	0.86
258610_at	dawn-box_ERS2	0.00	dawn-spike_GAOX6	0.00	dawn-box_ERS2	0.000	dawn-box_ERS2	0.34	dark-box_TIR1	0.00	dawn-spike_AC8	0.03
258818_at	*dawn-box_ERS2*	*0.01*	*dawn-box_ERS2*	*0.90*	*dawn-box_ERS2*	*0.001*	*dawn-box_ERS2*	*0.17*	*dark-box_TIR1*	*0.63*	*dawn-box_ERS2*	*0.23*
259042_at	dawn-spike_GAOX6	0.57	dawn-spike_AC8	0.82	dawn-spike_AC8	0.804	dawn-spike_GAOX6	0.80	dawn-spike_AC8	0.03	dark-box_TIR1	0.27
259302_at	dawn-box_ERS2	0.23	dark-box_TIR1	0.00	dawn-box_ERS2	0.000	dawn-box_ERS2	0.18	dawn-box_ERS2	0.14	dawn-box_ERS2	0.06
259439_at	dawn-spike_GAOX6	0.10	dawn-spike_AC8	0.00	dawn-box_ERS2	0.481	dawn-spike_AC8	0.07	dawn-spike_AC8	0.02	dawn-spike_AC8	0.38
259445_at	dawn-spike_AC8	0.00	dawn-spike_GAOX6	0.00	dawn-box_ERS2	0.000	dawn-box_ERS2	0.03	dawn-spike_GAOX6	0.01	dawn-box_ERS2	0.01
259453_at	dawn-spike_AC8	0.39	dawn-box_ERS2	0.60	dawn-box_ERS2	0.922	dawn-spike_AC8	0.00	dawn-box_ERS2	0.62	dawn-spike_AC8	0.09
259466_at	dawn-box_ERS2	0.02	dawn-spike_AC8	0.47	dawn-spike_AC8	0.204	dawn-box_ERS2	0.46	dawn-spike_AC8	0.47	dawn-box_ERS2	0.41
259614_at	dark-box_TIR1	0.20	dawn-box_ERS2	0.23	dawn-spike_GAOX6	0.002	dawn-spike_AC8	0.00	dawn-spike_AC8	0.00	dawn-spike_AC8	0.37
259645_at	dawn-spike_AC8	0.00	dawn-spike_GAOX6	0.00	dawn-spike_AC8	0.000	dawn-spike_GAOX6	0.00	dawn-spike_AC8	0.00	dawn-spike_AC8	0.00
259669_at	dawn-spike_GAOX6	0.00	dawn-spike_AC8	0.91	dark-box_TIR1	0.126	dawn-box_ERS2	0.98	dawn-spike_AC8	0.42	dark-box_TIR1	0.66
259714_at	dawn-spike_AC8	0.40	dawn-spike_GAOX6	0.00	dark-box_TIR1	0.002	dawn-box_ERS2	0.00	dawn-box_ERS2	0.09	dawn-spike_GAOX6	0.07
259955_s_at	*dawn-box_ERS2*	*0.00*	*dawn-box_ERS2*	*0.57*	*dawn-box_ERS2*	*0.000*	*dawn-box_ERS2*	*0.00*	*dawn-box_ERS2*	*0.00*	*dawn-box_ERS2*	*0.00*
260023_at	dawn-box_ERS2	0.00	dawn-spike_GAOX6	0.00	dawn-spike_AC8	0.869	dark-box_TIR1	0.00	dawn-spike_AC8	0.49	dawn-spike_AC8	0.35
260133_at	dark-box_TIR1	0.01	dark-box_TIR1	0.00	dawn-box_ERS2	0.011	dawn-box_ERS2	0.33	dark-box_TIR1	0.00	dark-box_TIR1	0.00
260141_at	dark-box_TIR1	0.00	dawn-spike_AC8	0.00	dawn-box_ERS2	0.239	dawn-spike_GAOX6	0.00	dawn-box_ERS2	0.10	dawn-box_ERS2	0.01
260152_at	dawn-spike_AC8	0.00	dawn-spike_GAOX6	0.00	dawn-spike_AC8	0.000	dawn-spike_GAOX6	0.00	dawn-box_ERS2	0.03	dawn-spike_AC8	0.00
260240_at	*dawn-spike_GAOX6*	*0.19*	*dawn-spike_GAOX6*	*0.73*	*dawn-spike_GAOX6*	*0.002*	*dawn-spike_GAOX6*	*0.00*	*dawn-spike_AC8*	*0.24*	*dawn-spike_AC8*	*0.01*
260299_at	dawn-spike_AC8	0.85	dawn-box_ERS2	0.43	dawn-spike_GAOX6	0.000	dawn-spike_AC8	0.00	dawn-box_ERS2	0.45	dawn-spike_AC8	0.42
260300_at	dawn-spike_GAOX6	0.72	dawn-spike_AC8	0.82	dawn-spike_AC8	0.755	dawn-spike_AC8	0.60	dawn-box_ERS2	0.93	dawn-spike_AC8	0.63
260345_at	dark-box_TIR1	0.00	dark-box_TIR1	0.00	dawn-box_ERS2	0.000	dawn-box_ERS2	0.00	dawn-spike_GAOX6	0.00	dawn-box_ERS2	0.00
260637_at	dark-box_TIR1	0.00	dark-box_TIR1	0.00	dawn-box_ERS2	0.000	dawn-spike_AC8	0.04	dawn-box_ERS2	0.00	dark-box_TIR1	0.00
260773_at	dawn-spike_GAOX6	0.70	dawn-box_ERS2	0.77	dawn-box_ERS2	0.890	dawn-spike_GAOX6	0.76	dawn-spike_GAOX6	0.73	dawn-spike_GAOX6	0.52
260797_at	dawn-spike_GAOX6	0.67	dawn-box_ERS2	0.41	dawn-box_ERS2	0.147	dawn-spike_GAOX6	0.00	dawn-box_ERS2	0.01	dawn-spike_AC8	0.01
261109_at	dawn-spike_AC8	0.00	dawn-box_ERS2	0.13	dawn-spike_AC8	0.002	dawn-spike_AC8	0.00	dark-box_TIR1	0.04	dawn-spike_AC8	0.00
261521_at	dawn-box_ERS2	0.14	dawn-spike_AC8	0.95	dark-box_TIR1	0.625	dawn-box_ERS2	0.45	dawn-spike_AC8	0.91	dawn-box_ERS2	0.37
261768_at	dawn-spike_GAOX6	0.00	dawn-spike_GAOX6	0.73	dawn-spike_AC8	0.533	dawn-spike_GAOX6	0.00	dawn-spike_GAOX6	0.61	dawn-spike_GAOX6	0.68
261865_at	dawn-box_ERS2	0.03	dawn-box_ERS2	0.07	dawn-spike_AC8	0.285	dark-box_TIR1	0.50	dawn-box_ERS2	0.30	dawn-box_ERS2	0.96
262212_at	*dawn-box_ERS2*	*0.02*	*dawn-spike_AC8*	*0.80*	*dark-box_TIR1*	*0.000*	*dawn-box_ERS2*	*0.27*	*dawn-spike_AC8*	*0.83*	*dawn-box_ERS2*	*0.05*

Table S2. Phytohormone genes that match growth-associated models

262525_at	dark-box_TIR1	0.00	dark-box_TIR1	0.00	dawn-spike_GAOX6	0.000	dark-box_TIR1	0.02	dark-box_TIR1	0.00	dawn-spike_AC8	0.00
262850_at	dawn-spike_AC8	0.00	dawn-box_ERS2	0.00	dawn-spike_GAOX6	0.001	dark-box_TIR1	0.00	dark-box_TIR1	0.00	dawn-spike_AC8	0.01
262891_at	dawn-spike_AC8	0.98	dawn-box_ERS2	0.08	dawn-box_ERS2	0.966	dawn-box_ERS2	0.73	dawn-spike_AC8	0.24	dawn-spike_AC8	0.80
263170_at	dawn-spike_AC8	0.44	dawn-box_ERS2	0.00	dawn-spike_AC8	0.066	dawn-box_ERS2	0.31	dawn-spike_AC8	0.59	dawn-box_ERS2	0.29
263236_at	dawn-box_ERS2	0.01	dawn-spike_AC8	0.75	dawn-box_ERS2	0.862	dawn-box_ERS2	0.42	dawn-spike_AC8	0.81	dawn-box_ERS2	0.47
263241_at	dark-box_TIR1	0.11	dawn-spike_AC8	0.87	dawn-spike_AC8	0.760	dawn-box_ERS2	0.74	dawn-spike_AC8	0.77	dawn-spike_AC8	0.20
263377_at	dawn-spike_GAOX6	0.21	dawn-spike_GAOX6	0.00	dawn-spike_AC8	0.001	dawn-spike_AC8	0.00	dawn-spike_GAOX6	0.00	dawn-box_ERS2	0.02
263570_at	dawn-spike_GAOX6	0.77	dawn-spike_AC8	0.02	dark-box_TIR1	0.096	dawn-spike_AC8	0.45	dawn-spike_AC8	0.02	dawn-box_ERS2	0.68
263599_at	dawn-box_ERS2	0.02	dawn-spike_AC8	0.88	dark-box_TIR1	0.080	dawn-box_ERS2	0.34	dawn-spike_AC8	0.26	dawn-spike_AC8	0.87
263653_at	dawn-box_ERS2	0.02	dark-box_TIR1	0.00	dawn-box_ERS2	0.000	dawn-spike_GAOX6	0.00	dawn-box_ERS2	0.00	dawn-box_ERS2	0.00
263656_at	dawn-spike_GAOX6	0.00	dawn-box_ERS2	0.00	dawn-spike_AC8	0.000	dark-box_TIR1	0.01	dawn-box_ERS2	0.00	dawn-spike_GAOX6	0.11
263714_at	dawn-spike_AC8	0.96	dawn-spike_AC8	0.88	dawn-spike_AC8	0.948	dawn-box_ERS2	0.91	dawn-spike_AC8	0.96	dawn-spike_AC8	0.95
263907_at	dawn-spike_AC8	0.88	dawn-spike_GAOX6	0.00	dawn-box_ERS2	0.866	dawn-spike_AC8	0.53	dawn-box_ERS2	0.47	dawn-spike_AC8	0.01
264248_at	dawn-spike_AC8	0.00	dawn-box_ERS2	0.00	dawn-spike_AC8	0.000	dawn-spike_GAOX6	0.00	dawn-spike_GAOX6	0.00	dawn-spike_AC8	0.00
264448_at	dark-box_TIR1	0.27	dark-box_TIR1	0.01	dark-box_TIR1	0.000	dawn-box_ERS2	0.00	dark-box_TIR1	0.00	dark-box_TIR1	0.00
264470_at	dawn-box_ERS2	0.00	dawn-spike_GAOX6	0.74	dawn-spike_AC8	0.970	dawn-spike_GAOX6	0.61	dawn-box_ERS2	0.68	dawn-box_ERS2	0.66
264537_at	dawn-box_ERS2	0.00	dawn-spike_AC8	0.00	dark-box_TIR1	0.000	dark-box_TIR1	0.00	dawn-box_ERS2	0.64	dawn-box_ERS2	0.15
264586_at	dawn-box_ERS2	0.02	dark-box_TIR1	0.00	dawn-box_ERS2	0.000	dawn-spike_GAOX6	0.00	dawn-spike_GAOX6	0.00	dark-box_TIR1	0.00
264593_at	dawn-box_ERS2	0.47	dawn-spike_AC8	0.92	dawn-spike_AC8	0.862	dark-box_TIR1	0.05	dawn-box_ERS2	0.88	dawn-spike_AC8	0.68
264623_at	dawn-box_ERS2	0.57	dawn-box_ERS2	0.04	dawn-box_ERS2	0.381	dawn-spike_GAOX6	0.18	dawn-spike_GAOX6	0.31	dawn-spike_GAOX6	0.78
264823_at	dawn-box_ERS2	0.20	dawn-spike_AC8	0.09	dawn-box_ERS2	0.446	dawn-spike_AC8	0.00	dawn-box_ERS2	0.27	dawn-spike_AC8	0.02
264843_at	dawn-spike_GAOX6	0.00	dark-box_TIR1	0.00	dawn-spike_GAOX6	0.003	dark-box_TIR1	0.73	dark-box_TIR1	0.00	dawn-spike_GAOX6	0.00
265194_at	dawn-box_ERS2	0.01	dawn-box_ERS2	0.00	dawn-box_ERS2	0.000	dawn-spike_AC8	0.03	dawn-box_ERS2	0.00	dawn-box_ERS2	0.00
265200_s_at	dawn-spike_AC8	0.84	dawn-spike_AC8	0.61	dark-box_TIR1	0.145	dawn-spike_GAOX6	0.00	dark-box_TIR1	0.73	dawn-spike_GAOX6	0.60
265250_at	*dawn-box_ERS2*	*0.00*	*dawn-spike_AC8*	*0.84*	*dark-box_TIR1*	*0.000*	*dawn-box_ERS2*	*0.15*	*dawn-spike_AC8*	*0.02*	*dark-box_TIR1*	*0.00*
265615_at	dawn-box_ERS2	0.22	dawn-spike_AC8	0.01	dawn-spike_GAOX6	0.001	dawn-spike_AC8	0.95	dawn-spike_AC8	0.77	dawn-spike_AC8	0.95
265633_at	dawn-box_ERS2	0.24	dawn-box_ERS2	0.00	dawn-box_ERS2	0.000	dawn-box_ERS2	0.01	dawn-box_ERS2	0.00	dawn-box_ERS2	0.00
265945_at	dark-box_TIR1	0.15	dawn-box_ERS2	0.66	dawn-spike_AC8	0.610	dawn-spike_GAOX6	0.00	dawn-spike_AC8	0.30	dawn-spike_AC8	0.00
265948_at	dawn-spike_AC8	0.69	dawn-spike_AC8	0.10	dawn-spike_AC8	0.836	dawn-spike_AC8	0.07	dawn-spike_AC8	0.85	dawn-spike_AC8	0.98
266078_at	dawn-box_ERS2	0.00	dawn-box_ERS2	0.12	dawn-box_ERS2	0.000	dawn-box_ERS2	0.05	dawn-box_ERS2	0.11	dawn-box_ERS2	0.00
266095_at	dawn-spike_GAOX6	0.85	dark-box_TIR1	0.00	dawn-box_ERS2	0.042	dawn-box_ERS2	0.91	dawn-box_ERS2	0.00	dawn-spike_AC8	0.02
266302_at	dark-box_TIR1	0.00	dawn-box_ERS2	0.00	dark-box_TIR1	0.001	dark-box_TIR1	0.00	dark-box_TIR1	0.00	dark-box_TIR1	0.00
266331_at	dawn-spike_GAOX6	0.00	dark-box_TIR1	0.09	dark-box_TIR1	0.006	dawn-spike_GAOX6	0.00	dawn-spike_AC8	0.72	dawn-spike_GAOX6	0.00
266335_at	dawn-spike_AC8	0.03	dawn-box_ERS2	0.00	dawn-spike_AC8	0.000	dawn-spike_AC8	0.16	dawn-box_ERS2	0.01	dawn-spike_GAOX6	0.64
266372_at	dark-box_TIR1	0.06	dawn-spike_AC8	0.92	dawn-spike_AC8	0.573	dawn-box_ERS2	0.41	dark-box_TIR1	0.78	dark-box_TIR1	0.04
266778_at	dawn-spike_AC8	0.86	dawn-spike_AC8	0.29	dawn-spike_AC8	0.940	dawn-spike_AC8	0.56	dark-box_TIR1	0.09	dawn-spike_GAOX6	0.80

Table S2. Phytohormone genes that match growth-associated models

266830_at	dawn-spike_AC8	0.00	dawn-box_ERS2	0.00	dawn-spike_AC8	0.578	dawn-spike_AC8	0.01	dawn-spike_AC8	0.03	dawn-spike_AC8	0.84
266995_at	dawn-spike_GAOX6	0.00	dawn-spike_AC8	0.00	dawn-spike_GAOX6	0.000	dawn-spike_GAOX6	0.01	dawn-spike_AC8	0.00	dawn-spike_GAOX6	0.05
267614_at	dawn-spike_AC8	0.00	dark-box_TIR1	0.00	dawn-spike_GAOX6	0.000	dark-box_TIR1	0.00	dawn-spike_GAOX6	0.00	dawn-spike_AC8	0.00
