# Supplementary material for: A Morning-Specific Phytohormone Gene Expression Program underlying Rhythmic Plant Growth
Source: PLoS Biol. 2008 Sep 16;6(9):e225. doi: 10.1371/journal.pbio.0060225 (PMC2535664; doi:10.1371/journal.pbio.0060225)
Supplement: Supplementary file 6 [file pbio.0060225.st003.pdf]

Table S3. 3-8mer words overrepresented in the 71 phytohormone gene promoters (500 bp).

word	count	zscore	onet p-vals	corrected p-val	hits
GCGTACGC	2	10.22	7.9967E-25	3.51E-20	1
CAGTCAGG	3	7.36	9.3544E-14	2.05E-09	3
CCGTGGTA	3	6.98	1.485E-12	2.17E-08	3
CCACATGG	***6***	***6.88***	***2.9361E-12***	***3.22E-08***	***6***
GATAGCGC	2	6.87	3.17E-12	2.78E-08	2
CGAGCCCC	2	6.46	5.1696E-11	3.24E-07	2
ACGGCTGG	3	6.45	5.6246E-11	3.08E-07	2
GGTCATAC	4	6.42	6.9635E-11	3.39E-07	4
CGGTCACA	4	6.42	6.9635E-11	3.05E-07	4
GCGTGACC	3	6.40	7.5857E-11	3.02E-07	2
CGCGTAAG	3	6.38	9.0743E-11	3.32E-07	3
AAGCCGCG	3	6.38	9.0743E-11	3.06E-07	3
ACTATAGT	14	6.33	1.2189E-10	3.82E-07	4
ACGAGGCA	4	6.29	1.6171E-10	4.73E-07	4
ATCGCGGC	2	6.22	2.4447E-10	6.70E-07	2
ATTGCGGG	2	6.22	2.4447E-10	6.31E-07	2
CTGTGCGC	1	6.16	3.6213E-10	8.82E-07	1
GGAGGGCC	2	6.01	9.5382E-10	2.20E-06	2
AATGCGCT	3	5.95	1.3549E-09	2.97E-06	3
GAAGCCGC	3	5.90	1.8206E-09	3.80E-06	3
CCGACAGG	2	5.63	9.2661E-09	1.85E-05	2
ATACATAT	20	5.60	1.0718E-08	2.04E-05	13
GGCATACC	2	5.46	2.4184E-08	4.24E-05	2
GATGGGGC	2	5.46	2.4184E-08	4.08E-05	2
ACTATA	66	5.41	3.2336E-08	5.25E-05	40
GCCGACCC	2	5.38	3.7672E-08	5.90E-05	2
CTGCCCCC	2	5.38	3.7672E-08	5.70E-05	2
GACAGGTC	3	5.35	4.4957E-08	6.57E-05	3
CTCACGTA	4	5.33	4.96E-08	7.02E-05	4
CACATGGA	***6***	***5.30***	***5.6951E-08***	***7.80E-05***	***6***
ACGCCCGG	1	5.20	9.7749E-08	0.000129876	1
ACCCCGCG	1	5.20	9.7749E-08	0.000126056	1
CACATG	***41***	***5.20***	***9.8221E-08***	***0.000123046***	***32***
CACATGG	***12***	***5.20***	***9.8863E-08***	***0.00012041***	***12***
CCCCACA	9	5.18	1.0859E-07	0.000128682	9
ACGTACTA	4	5.18	1.132E-07	0.000130615	3
ATAGCGCG	2	5.17	1.1483E-07	0.000129098	2
CACGGCTG	2	5.16	1.2566E-07	0.000137742	2
ACCGTATG	3	5.10	1.7353E-07	0.000185576	3
ATGTGGGG	4	5.09	1.7816E-07	0.000185991	4
CCTTACGC	2	5.09	1.8096E-07	0.00018452	2
AGAGAGAC	15	5.08	1.9255E-07	0.000191876	11
CTACAGCA	3	5.06	0.000000207	0.000201692	3
GAGAGACA	11	4.99	3.0242E-07	0.000288259	10
ATAGGTGG	4	4.99	3.0903E-07	0.000288292	4
ACAGTATC	5	4.97	3.2748E-07	0.000299139	5
AGGAAAGG	7	4.95	3.7248E-07	0.000333301	5
AGCCGTG	5	4.94	3.9164E-07	0.000343437	5

Table S3. 3-8mer words overrepresented in the 71 phytohormone gene promoters (500 bp).

GTGACCGA	3	4.93	4.0416E-07	0.000347467	3
ACATGGGA	5	4.92	4.3492E-07	0.000366721	5
CACGCTAC	2	4.89	4.9203E-07	0.000407048	2
TACTATGA	7	4.85	6.0677E-07	0.000492675	7
GTGGTACA	4	4.83	6.7747E-07	0.000540079	4
CATACACC	4	4.83	6.9873E-07	0.000547081	4
CTTGCCTC	4	4.82	7.0614E-07	0.000543183	3
CCGCACCG	2	4.81	7.6482E-07	0.000578178	2
GGTCTCTA	4	4.76	9.7617E-07	0.000725443	4
CCAGGCGG	1	4.76	9.886E-07	0.000722436	1
GCCCCGTA	1	4.76	9.886E-07	0.000710593	1
ATGTGAGC	4	4.72	1.1645E-06	0.000823527	4
CAATGCGC	2	4.72	1.1857E-06	0.00082521	2
AGGTCCCG	2	4.72	1.1857E-06	0.000812316	2
ACTATAAC	7	4.72	1.1946E-06	0.000805822	7
AGAGACA	24	4.72	1.1985E-06	0.000796204	19
ATACTGTG	4	4.71	1.2119E-06	0.000793089	4
AATCTCAC	9	4.69	1.3602E-06	0.000877049	8
TACATATA	19	4.67	1.4787E-06	0.000939639	13
CGACAGGA	2	4.66	1.5533E-06	0.000972943	2
GCCTCTAA	4	4.65	1.6376E-06	0.001011299	4
TGCGATAA	4	4.64	1.7279E-06	0.001052243	4
CCATCCGG	2	4.63	1.8708E-06	0.001123659	2
CATGTGG	12	4.62	1.9234E-06	0.00113964	11
CCCCTTGC	2	4.61	2.0139E-06	0.001177353	2
GAGCCCCA	2	4.61	2.0139E-06	0.001161861	2
AAAGCACG	4	4.60	2.0852E-06	0.001187372	4
AATGCGTA	4	4.60	2.0941E-06	0.001177153	4
CGCGGTGC	1	4.57	2.4634E-06	0.001367218	1
CGCTATAC	2	4.56	2.5854E-06	0.001416993	2
ATCGCGTA	2	4.56	2.5854E-06	0.001399499	2
CTCTCCCC	4	4.54	2.7582E-06	0.00147483	3
CTATA	148	4.51	3.2031E-06	0.001692086	59
GAGGCAA	10	4.51	3.2113E-06	0.001676222	8
AGCCGTGG	2	4.51	3.2886E-06	0.001696376	2
CGTGACCC	2	4.51	3.2886E-06	0.001676651	2
AGAGGGGG	3	4.49	3.6452E-06	0.001837097	3
CATAGTAG	4	4.46	0.000004181	0.002083183	4
AGAGAC	46	4.44	4.4832E-06	0.002208656	28
GTGTCCCA	3	4.42	4.9359E-06	0.002404661	3
CACCTTAC	3	4.42	4.9359E-06	0.002378236	3
CTATAGTA	7	4.42	4.9616E-06	0.002364634	4
GGGGCAGA	2	4.41	0.000005185	0.002418527	2
CGGCTGGA	2	4.41	0.000005185	0.002444532	2
CGGTCCCA	2	4.41	0.000005185	0.002393069	2
AGCCCCAT	2	4.41	0.000005185	0.002368141	2
CCGCGCAC	1	4.40	5.4466E-06	0.002461976	1
GCGGCCCC	1	4.40	5.4466E-06	0.002436853	1
GAAGGGTC	3	4.40	5.5194E-06	0.002444481	3

Table S3. 3-8mer words overrepresented in the 71 phytohormone gene promoters (500 bp).

AGCCGCGA	2	4.39	5.6262E-06	0.002466864	2
AATGGGGG	3	4.39	5.7803E-06	0.002509337	3
GTCTCTA	13	4.36	6.4182E-06	0.002758945	10
GACGCGGC	2	4.36	0.000006433	0.002738459	2
ATATACAT	18	4.31	8.1856E-06	0.003451017	13
CTATCAGA	4	4.30	8.3839E-06	0.003500957	3
CCACGTGC	3	4.30	8.5931E-06	0.003554463	2
CAATCCGA	4	4.29	8.9772E-06	0.003644577	4
ACATGGG	***9***	***4.28***	***9.4641E-06***	***0.003806999***	***9***
CCAGTCAG	2	4.27	9.6878E-06	0.003861557	2
GAAGCCAC	4	4.26	0.000010194	0.004026722	3
ATACAGCG	2	4.24	0.000011045	0.00432392	2
AAATAAAA	61	4.24	0.000011164	0.00433183	39
TACGCGA	4	4.23	0.000011922	0.004545496	4
CACATGGG	***4***	***4.22***	***0.000012372***	***0.004676403***	***4***
GAGCCCC	3	4.21	0.000012655	0.004742488	3
GCGATAA	7	4.19	0.000013644	0.005069787	7
CACGTGGG	4	4.19	0.000014067	0.005183039	4
ATCACGGG	2	4.19	0.000014203	0.005189539	2
ACTATACT	7	4.19	0.000014231	0.005156797	6
CCCCAC	16	4.18	0.000014382	0.005168796	13
GTGGGGGA	3	4.18	0.000014542	0.005183809	3
CAGTGAAG	5	4.18	0.000014542	0.005142004	5
CCCCACCA	4	4.16	0.000015816	0.005547747	3
GTAAGCAC	3	4.15	0.000016821	0.005853441	3
GGAAAGGA	6	4.15	0.000016953	0.005852923	6
ATGGGCGG	2	4.14	0.00001704	0.005836999	2
ATAGTA	53	4.13	0.000018001	0.006118386	35
ATCACGG	6	4.13	0.000018487	0.006235238	6
GTCGGTCA	3	4.11	0.000020187	0.006705449	3
AGTAGTAG	8	4.10	0.000020238	0.006622055	5
AGTAAATA	13	4.09	0.000021394	0.006948454	12
AACATGGC	4	4.07	0.000023052	0.007431897	4
AGGCAAGG	3	4.06	0.000024095	0.007711455	3
CAATGGGG	3	4.06	0.000024095	0.007655575	3
GAGGTCCC	2	4.06	0.000024111	0.007605546	2
GCTGTAGA	3	4.05	0.000025091	0.007858143	3
CGCTACTA	2	4.03	0.000028453	0.008847874	2
GCCCTACA	2	4.03	0.000028453	0.008785565	2
AACCGTAG	3	4.02	0.000028608	0.008771653	3
ATGTGACC	4	4.02	0.000028789	0.008765851	4
TCGAGGAA	5	4.01	0.00002986	0.008967408	5
CCTTTGTC	5	4.00	0.000031049	0.009261051	5
GTACATCA	4	4.00	0.000031114	0.009217733	3
GTAGAGAC	4	4.00	0.000032077	0.009439249	4
GTGTGGTA	4	3.99	0.000033067	0.009665705	4
TCGCGTAA	2	3.99	0.000033409	0.009701	2
TACCGACA	3	3.98	0.000033797	0.0097491	3
ATCTCAC	14	3.98	0.000034653	0.009930689	13

Table S3. 3-8mer words overrepresented in the 71 phytohormone gene promoters (500 bp).

GTAAGAAA	13	3.96	0.000037424	0.010451546	10
ATAGCGC	3	3.95	0.000038407	0.010658186	3
ATGAGGGG	2	3.95	0.000039042	0.010766261	2
ATAATAAT	35	3.95	0.000039344	0.010781731	22
ACATGG	27	3.94	0.000040941	0.011149684	24
CAACCGTA	3	3.94	0.000041298	0.011177482	3
AATAAAA	97	3.93	0.000041863	0.01126089	53
ATGGGGGA	3	3.93	0.00004301	0.011498881	3
TAATAA	132	3.92	0.000043692	0.011610421	55
CGTATACG	4	3.89	0.000049809	0.013156177	2
ACTAT	137	3.88	0.000051655	0.013562067	58
TCTCCCCA	4	3.88	0.000052039	0.013581559	3
ACGAATAG	4	3.88	0.000052497	0.01362002	4
AGAATCAC	6	3.87	0.000054375	0.014024272	6
TAGAGAGA	15	3.87	0.000054818	0.014055848	10
ATCACG	20	3.87	0.000054976	0.014014405	14
CGTGCATC	2	3.86	0.000057499	0.014324438	2
GGTTGCTA	3	3.84	0.000060603	0.015012425	3
GAGAGAC	18	3.82	0.000065656	0.01617277	14
ACTATGAC	4	3.82	0.000068079	0.016675932	4
ATATGTA	31	3.81	0.000068873	0.016776698	20
GGGCTCGA	2	3.81	0.000069704	0.016885313	2
CTCCAGCC	2	3.81	0.000069704	0.016792536	2
TAGTAATA	11	3.79	0.000074158	0.017767933	10
ATAATAA	63	3.79	0.000074552	0.017765255	33
AGGCTCAC	2	3.78	0.000079771	0.018906158	2
CATGTGAC	5	3.77	0.000080199	0.018905405	5
CGAGGCA	4	3.76	0.000083434	0.019562819	4
ACCAGCTC	3	3.76	0.000083446	0.01946156	3
ACAAAGGA	8	3.76	0.000085341	0.019798209	8
CCAGGATC	2	3.76	0.0000864	0.019938392	2
CGGTCAC	5	3.76	0.000086586	0.0198767	5
AACAACGC	3	3.75	0.000086672	0.019792815	3
CCTGCTTC	3	3.75	0.000086672	0.019690262	3
AGAGGCAA	5	3.74	0.000092196	0.020624622	4
ATTATTA	59	3.72	0.000099664	0.02218207	34
CCCCCAC	5	3.72	0.00010119	0.022407963	5
CTACCGAC	2	3.71	0.00010335	0.022771277	2
GCGTTGTA	2	3.71	0.00010335	0.022657421	2
CATTACAA	9	3.71	0.00010337	0.02254906	8
CCCTTGCC	2	3.70	0.00010589	0.02298442	2
TCCTCGAA	4	3.68	0.00011661	0.025186611	4
ACGCTACT	2	3.67	0.00011969	0.025725136	2
ACATCATA	7	3.67	0.00012116	0.025914055	6
ACATG	118	3.67	0.00012259	0.026092627	54
AGGACAAA	7	3.66	0.0001264	0.026773596	7
ATAAAAT	69	3.66	0.00012811	0.027005342	43
ATCTCACG	4	3.65	0.00012887	0.027035569	4
ATACATA	30	3.65	0.00013125	0.02740375	21

Table S3. 3-8mer words overrepresented in the 71 phytohormone gene promoters (500 bp).

ACTTGAGC	4	3.65	0.00013232	0.027496221	4
ACCACGG	4	3.65	0.00013285	0.027476137	4
ATGCCCAT	3	3.64	0.00013762	0.028196666	3
TAGGGTCA	3	3.62	0.00014502	0.029574637	3
GTAAATAA	14	3.62	0.00014697	0.029833549	12
AAAATAAA	58	3.62	0.00014885	0.030075931	33
TAATAGTA	10	3.62	0.00014898	0.029964115	9
AAGTAGTA	7	3.61	0.000154	0.0306922	7
TACTA	131	3.61	0.00015527	0.030805287	57
GGCCGCC	3	3.60	0.00016029	0.031657997	3
GGGGAC	10	3.59	0.00016313	0.03207443	9
AGACTATA	6	3.59	0.00016467	0.032232682	6
ATGCTCC	6	3.59	0.00016527	0.032206349	5
CTCTGCCC	2	3.59	0.00016675	0.032350976	2
CCGCTGAA	2	3.59	0.00016675	0.03220846	2
GACATGCA	4	3.58	0.00017128	0.03279451	3
ACGAATA	13	3.57	0.00017553	0.033462123	12
GACGGACA	2	3.57	0.00017793	0.033772809	2
GTCCTTAA	4	3.56	0.00018526	0.035012543	4
AATCACGG	3	3.56	0.00018566	0.034937547	3
AGATACGG	3	3.56	0.00018683	0.035007471	3
AGTGGGAC	3	3.56	0.00018683	0.034858503	3
ATACCGCG	2	3.55	0.00019141	0.035411658	2
GGTAGAAA	5	3.54	0.0001967	0.035202074	5
GGTCCCAC	3	3.54	0.00019687	0.035089277	3
TCTGATAA	7	3.54	0.0002011	0.0356981	6
ATCGCGG	3	3.54	0.00020335	0.035951952	3
TAGGAGAA	6	3.53	0.00020523	0.036138613	6
ACCATTAC	5	3.53	0.00020523	0.035994058	4
TCGTGGCA	2	3.53	0.00020808	0.036348509	2
ATGCTCCC	2	3.53	0.00020808	0.036204269	2
CTATACCA	4	3.53	0.00020836	0.036109694	4
AGAGACAT	7	3.53	0.00021014	0.036274797	7
ATACA	163	3.52	0.00021318	0.036655256	63
CTTACGC	5	3.52	0.00021496	0.036816938	4
ATAAATAG	13	3.52	0.00021654	0.036943241	10
ACTATAA	22	3.52	0.00021841	0.036974536	21
TACCCGTA	2	3.51	0.0002211	0.037285964	2
CGGCTTC	5	3.51	0.00022394	0.037620204	5
GTGCTCAA	3	3.51	0.00022805	0.038164429	3
ATACTATC	5	3.50	0.00022897	0.038172694	5
CATATACG	4	3.50	0.00022997	0.038050055	4
AAGCCGTG	2	3.50	0.00023151	0.038160855	2
AACCGTAT	4	3.50	0.00023282	0.038233055	3
AGGGTCA	6	3.50	0.00023699	0.038772625	5
GGCTAGGA	2	3.49	0.0002455	0.040015587	2
AGACAGGC	2	3.49	0.0002455	0.039867381	2
CTCGCACA	2	3.49	0.0002455	0.039720269	2
AACACGGA	3	3.48	0.00025505	0.040813585	3

Table S3. 3-8mer words overrepresented in the 71 phytohormone gene promoters (500 bp).

AGACACCG	2	3.47	0.0002569	0.040960136	2
CAACTGGC	2	3.47	0.0002569	0.04081173	2
GTCCCAC	6	3.47	0.00026365	0.041732844	5
GTCCACAC	3	3.46	0.00026732	0.042161557	3
ACCACGGT	2	3.46	0.00027489	0.042142752	2
CGGTCA	14	3.45	0.00027961	0.042717004	13
GGTAGAA	9	3.45	0.00028321	0.043116756	9
CGTGGTAG	2	3.45	0.00028437	0.043143554	2
CCATTAC	11	3.45	0.00028548	0.043162607	10
ATAAAA	182	3.44	0.00029068	0.043797784	67
CCTGTCG	3	3.44	0.00029105	0.04370335	3
ATACTTGA	6	3.44	0.00029486	0.04412434	6
ATTTTA	159	3.43	0.00029881	0.044563344	59
AAGGACGG	2	3.43	0.00030045	0.044656036	2
ACCGACAG	2	3.43	0.00030045	0.044505171	2
ACAACGC	5	3.43	0.00030333	0.044780496	5
AACGAATA	7	3.41	0.00032429	0.047714159	6
TAATAATA	30	3.40	0.00033181	0.048495138	19
CCGTCC	10	3.40	0.00033242	0.048422881	10
AATTATAT	22	3.40	0.00033707	0.048937653	17
AAAATCTT	19	3.40	0.00034273	0.049269966	16
CCTTGCAG	2	3.39	0.00034599	0.04957607	2
ACACGCTA	2	3.39	0.00034599	0.049414585	2
ACACCGAG	2	3.39	0.00034599	0.049254148	2
CGAGGCAA	2	3.39	0.00034599	0.049094749	2
CGGTTGAC	2	3.39	0.00034599	0.048936379	2
AGTCCTCG	2	3.39	0.00034599	0.048779027	2
ATATGAAC	7	3.39	0.00034781	0.048878453	6
